# Does Bentonite Cause Cytotoxic and Whole-Transcriptomic Adverse Effects in Enterocytes When Used to Reduce Aflatoxin B1 Exposure?

**DOI:** 10.3390/toxins14070435

**Published:** 2022-06-26

**Authors:** Greta Mucignat, Irene Bassan, Mery Giantin, Marianna Pauletto, Anisa Bardhi, Silvia Iori, Rosa Maria Lopparelli, Andrea Barbarossa, Anna Zaghini, Enrico Novelli, Mauro Dacasto

**Affiliations:** 1 Department of Comparative Biomedicine and Food Science, University of Padua, Viale dell’Università 16, 35020 Legnaro, Padua, Italy; greta.mucignat@unipd.it (G.M.); irene.bassan@unipd.it (I.B.); mery.giantin@unipd.it (M.G.); marianna.pauletto@unipd.it (M.P.); silvia.iori@phd.unipd.it (S.I.); rosa.lopparelli@unipd.it (R.M.L.); enrico.novelli@unipd.it (E.N.); 2 Department of Veterinary Medical Sciences, Alma Mater Studiorum University of Bologna, Via Tolara di Sopra 50, 40064 Ozzano dell’Emilia, Bologna, Italy; anisa.bardhi@unibo.it (A.B.); andrea.barbarossa@unibo.it (A.B.); anna.zaghini@unibo.it (A.Z.)

**Keywords:** aflatoxin B1, bentonite, Caco-2, clays, detoxification, cytotoxicity, in vitro permeability, RNA-seq

## Abstract

Aflatoxin B1 (AFB1) is a major food safety concern, threatening the health of humans and animals. Bentonite (BEN) is an aluminosilicate clay used as a feed additive to reduce AFB1 presence in contaminated feedstuff. So far, few studies have characterized BEN toxicity and efficacy in vitro. In this study, cytotoxicity (WST-1 test), the effects on cell permeability (trans-epithelial electrical resistance and lucifer yellow dye incorporation), and transcriptional changes (RNA-seq) caused by BEN, AFB1 and their combination (AFB1 + BEN) were investigated in Caco-2 cells. Up to 0.1 mg/mL, BEN did not affect cell viability and permeability, but it reduced AFB1 cytotoxicity; however, at higher concentrations, BEN was cytotoxic. As to RNA-seq, 0.1 mg/mL BEN did not show effects on cell transcriptome, confirming that the interaction between BEN and AFB1 occurs in the medium. Data from AFB1 and AFB1 + BEN suggested AFB1 provoked most of the transcriptional changes, whereas BEN was preventive. The most interesting AFB1-targeted pathways for which BEN was effective were cell integrity, xenobiotic metabolism and transporters, basal metabolism, inflammation and immune response, p53 biological network, apoptosis and carcinogenesis. To our knowledge, this is the first study assessing the in vitro toxicity and whole-transcriptomic effects of BEN, alone or in the presence of AFB1.

## 1. Introduction

Aflatoxins (AFs) are a group of mycotoxins produced by the secondary metabolism of some fungal species such as *Aspergillus flavus* and *Aspergillus parasiticus* in particular conditions of temperature and humidity. Among the different AFs identified so far, five are considered relevant for their diffusion and toxicity: AFB1, AFB2, AFG1, AFG2 and AFM1. These mycotoxins can be found in important food commodities such as peanuts, millet, sesame seeds, maize, wheat, rice, figs and other dried fruit, spices, unrefined vegetable oils and cocoa beans due to pre- and/or post-harvest fungal infections [1]. Moreover, they can be found in milk and dairy products derived from dairy cows fed with contaminated feedstuffs. Indeed, AFs are only partially degraded by the ruminal flora, while the remaining fraction is absorbed by the digestive tract and hydroxylated in the liver to AFM1, which in turn can be excreted into milk [2,3,4]. Alarmingly, AFM1 has also been found in human breast milk, thus representing an important threat to breastfeeding newborn health [5].

Aflatoxin B1 has hepatotoxic, immunotoxic, mutagenic, carcinogenic and teratogenic properties in human and farm animals [6,7,8,9]. It has been associated with human hepatocellular carcinoma (HCC), and since 2012 the International Agency for Research on Cancer (IARC) has classified AFB1 and the other AFs as carcinogenic to humans (Group I) [10]. Once ingested, AFB1 is bioactivated in the liver by cytochromes P450 (CYPs) into different metabolites such as AFM1, aflatoxicol (AFL), AFB2a, AFQ1, AFP1 and the most toxic AFB1-exo-8,9-epoxide (AFBO). This latter derivative is extremely reactive and can bind to guanine in the DNA and to lysine residues in the proteins, leading to DNA mutation and protein damage [11]. Furthermore, it causes oxidative stress, immune system impairment, malnutrition, intestinal inflammatory diseases and growth impairment in humans as well as in farm animals [12,13,14,15,16].

Aflatoxins contamination represents a major issue from a health and economic standpoint. The consumption of AF-contaminated food and feedstuff can lead to acute and chronic toxic effects whose gravity depends on various factors such as species, age, sex and exposure level. In livestock farming, AF-contaminated feed leads to a decrease in animal productivity, growth and final product quality, thus negatively impacting the whole supply chain [17,18].

For these reasons, AF research focuses on two main strategies: limiting AFB1 production in the field and decreasing its presence in already contaminated feedstuff and derived products. To mitigate AFB1 negative effects, different measures can be undertaken before and after the harvest. Pre-harvest methods consist of the prevention with good agricultural, storage and manufacturing practices as well as biological control on the field (i.e., by taking advantage of the biofungicide characteristics of some specific microorganisms) [19]. Post-harvest methods consist of eliminating AFB1 from already contaminated feeds and foods. Decontamination occurs by removing the mycotoxin through physical (sorting, heating, irradiation and cold plasma treatment), chemical (ozonation, acids, bases, oxidizing agents and reducing agents) or biological (use of specific microorganisms that can transform mycotoxins into less toxic compounds) treatments. In addition, AFB1 decontamination can also be achieved by using adsorbent materials such as minerals, chemicals and organic adsorbents that, once added to the contaminated feedstuff, decrease the AFs bioavailability [19]. This latter is the AFs detoxification strategy most commonly used in animal husbandry, and bentonite (BEN) is the most widely used adsorbent mineral clay [20].

Bentonite is an adsorbent aluminosilicate clay consisting mostly of montmorillonite, commonly used as a feed additive to reduce AFs bioavailability (and, consequently, toxicity) in the gastrointestinal tract [18,21,22]. Experimental data show that AFM1 content in milk can be diminished by 60.4% when introducing 227 g bentonite/cow/day into cattle diet [23]. This clay is considered non-toxic, and its use of up to 2% as a feed additive is authorized by EFSA [24,25]; however, some in vitro and in vivo studies reported possible undesirable effects due to clay administration such as mineral and vitamin unbalances, interactions with veterinary drugs, intestinal toxicity, hepatic damage and decreased growth performances [26,27].

To shed light on BEN’s possible adverse effects, in this study, we investigated the in vitro effects of BEN, either alone or in the presence of AFB1, on human enterocytes; specifically, we assessed its cytotoxicity, the possible modulation of cell permeability (i.e., membrane integrity and trans-membrane transport) as well as its whole-transcriptomic effects. We chose the Caco-2 cell line, a well-established in vitro model of intestinal barrier widely used for the prediction of intestinal xenobiotic permeability and absorption [28,29,30,31,32,33,34]. Confirmatory analytical investigations on the capability of BEN to adsorb AFB1 and its metabolites AFM1 and AFL were carried out by using mass spectrometry.

The present study provides the scientific community with important and new toxicological data supporting BEN supplementation in feed and its efficacy in mitigating AFs absorption and toxicity.

## 2. Results

### 2.1. Cytochrome P450 3A4 Induction

A quantitative real-time RT-PCR (qPCR) assay confirmed the slight, albeit significant, increase in *CYP3A4* expression after 24 h since the induction treatment was applied (*p* < 0.05; Figure S1A). As a consequence, induced cells (IND) showed a higher susceptibility to AFB1 when compared to those non-induced (nIND); however, such difference was statistically significant (*p* < 0.05) only at the highest AFB1 concentration (i.e., 90 µM; Figure S1B).

### 2.2. Assessment of BEN and AFB1 Cytotoxicity as Single Agents or in Combination

Bentonite cytotoxicity was assessed in both IND and nIND cells to understand the possible modulatory role of *CYP3A4* up-regulation. Caco-2 differentiated cells were incubated for 48 h with increasing concentrations of BEN (0.005–1.2 mg/mL). The estimated half-maximal inhibitory concentration (IC_50_) values were comparable in both conditions and corresponded to 0.08 mg/mL and 0.09 mg/mL, respectively (Figure S2A,B).

With regard to AFB1, IND cells (i.e., showing a *CYP3A4* up-regulation) were exposed for 48 h to increasing AFB1 concentrations (range 0.2–90 µM). As a whole, AFB1 was poorly cytotoxic; as a consequence, it was not possible to build up a dose–response curve and define the corresponding IC_50_ value (Figure S2C).

**Figure 1:**
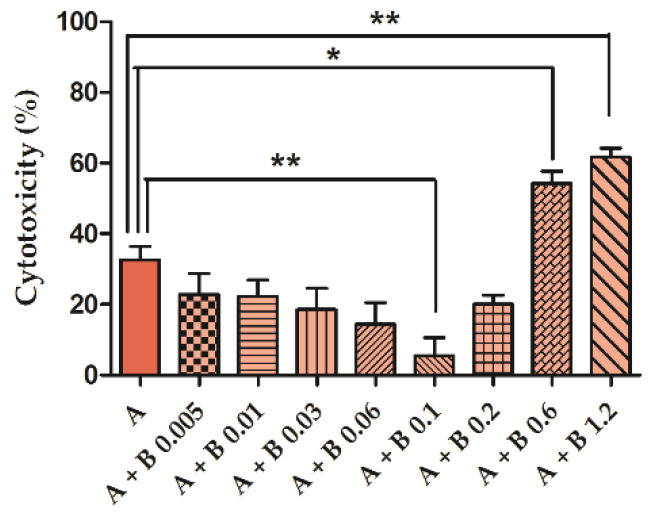
Aflatoxin B1 (AFB1, **A**) cytotoxicity in the presence of bentonite (BEN, **B**) increasing concentrations. Bars show the mortality of control differentiated Caco-2 cells treated for 48 h with 81 µM AFB1, alone or in combination with increasing BEN concentration (0.005–1.2 mg/mL). * *p* < 0.05; ** *p* < 0.01 (one-way ANOVA, followed by Bonferroni’s multiple comparison test). The graph shows only significant differences observed between treatments vs. control, for the benefit of the readers.

Once we defined the cytotoxicity of the two molecules taken individually, co-incubation experiments were executed using a fixed AFB1 concentration (81 µM) and BEN increasing concentrations. This co-incubation study showed that BEN might decrease AFB1 cytotoxicity in a dose-dependent manner and up to 0.1 mg/mL, where AFB1 mean cytotoxicity dropped from 32.6% to 5.5% (*p* < 0.01); however, BEN higher concentrations (0.6 and 1.2 mg/mL) significantly increase AFB1 cytotoxicity (*p* < 0.05 and *p* < 0.01, respectively; Figure 1).

As in our experimental conditions, the BEN concentration that most effectively reduced AFB1 cytotoxicity was 0.1 mg/mL; such a concentration was selected for the following experiments.

### 2.3. Evaluation of Caco-2 Monolayer Integrity Following the Exposure to BEN and AFB1, Either Alone or in Combination

Non-induced Caco-2 cell monolayers exposed to 0.1 mg/mL BEN did not show any alteration in both trans-epithelial electrical resistance (TEER) and paracellular permeability when compared to control cells (Figure S3A,B).

**Figure 2:**
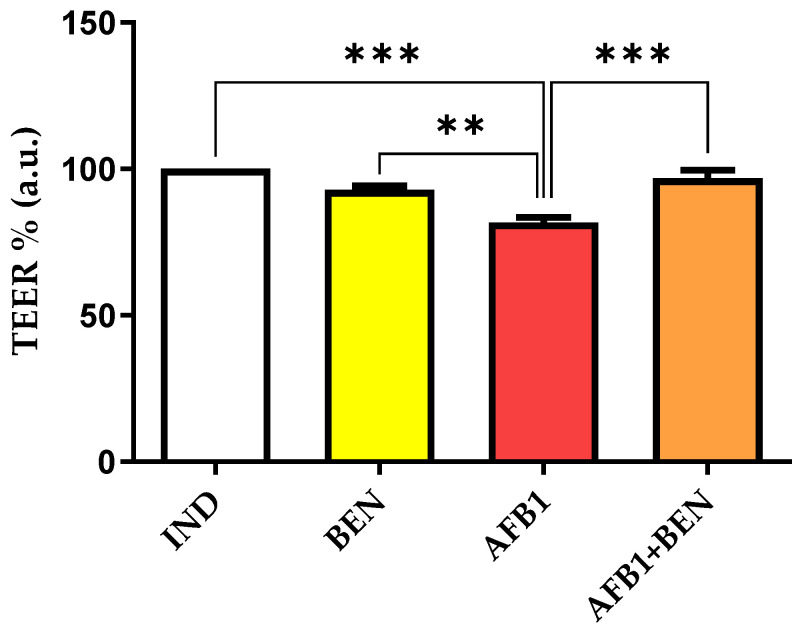
Effect of 0.1 mg/mL bentonite (BEN), aflatoxin B1 (AFB1) and the combination AFB1 + BEN on the trans-epithelial electrical resistance (TEER) in cytochrome P450 3A4 (*CYP3A4*)-induced Caco-2 cells (IND). Data (six independent cell culture experiments, each one run in duplicate) are reported as percentage of control cells (IND), whose value was set at 100%. **: *p* < 0.01; *** *p* < 0.001 (one-way ANOVA, followed by Bonferroni’s multiple comparison test).

Different results were observed in IND cells. Although no differences in TEER were noticed between BEN and controls, cells exposed to AFB1 showed a significant decrease in monolayer integrity (*p* < 0.001). It is worth noting that this effect was reverted when cells were exposed to BEN and AFB1 in combination (*p* < 0.001; Figure 2).

As for the permeability assay, no statistically significant results were ever observed (probably due to the variability of the data), even though the trend is visible in which cells exposed to AFB1 alone showed the highest lucifer yellow (LY) permeability and co-incubation with BEN and AFB1 brought the permeability back to values close to those of control cells (Figure S4).

It is noteworthy that a significant negative correlation was recorded in Caco-2 cells exposed to AFB1, between the values of TEER (i.e., a decreased monolayer resistance) and LY (i.e., an increased LY permeability; Pearson *r* = −0.95; *p* < 0.05).

### 2.4. LC-MS/MS Approaches to Assess the BEN Adsorbing Capacity and Its Effects on AFB1, AFM1, and AFL Transport

In the absence of cells, after 48 h of incubation at 37 °C, the mean percentage of free AFB1 in the cell medium containing BEN was 71.0 ± 2.1% (~29% reduction in the total amount of free AFB1).

As to the BEN adsorbent properties in the presence of the active monolayer, after 48 h of incubation at 37 °C the clay reduced the amount of free AFB1, AFM1 and AFL by ~42.0%, ~35.0% and ~50.0%, respectively (Table S1A).

Interestingly, the percentage of AFB1, AFM1 and AFL transport across the cellular membrane to the basolateral compartment were quite similar in cells exposed to AFB1 or AFB1 + BEN; however, AFM1 showed lower percentages (28.7% and 26.3%, respectively) compared to those obtained for AFB1 and AFL (~44.0% and ~45.5%, respectively; Table S1B). It is worth noting that the percentages we measured in AFB1 + BEN cells refer to the quantity of free AFB1 (i.e., not adsorbed by BEN), estimated to be ~58%, ~65%, and ~50% for AFB1, AFM1 and AFL, respectively (Table S1A).

### 2.5. Transcriptomic Effects of BEN and AFB1, Alone or in Combination

#### 2.5.1. Whole-Transcriptome Differential Expression Analysis

More than 34 million raw reads were obtained for each sample. After passing quality control, the trimming process allowed us to sort out 34,528,094 reads per sample (on average); after assessing the presence of no significant rRNA, the mean mapping percentage was 88.8% (Table S2).

The MultiDimensional Scaling (MDS) plot representing dimethyl sulfoxide (DMSO), nIND and BEN experimental groups showed no significant separation according to neither the first nor the second principal component; they represented a sort of homogenous group without significant differences in gene expression (data not shown). Such a finding was supported by the results of the Quasi-likelihood ratio F-test (QLF-test), which confirmed the absence of differential expressed genes (DEGs) among the aforementioned treatment groups (nIND, BEN, DMSO); therefore, we can affirm that BEN and DMSO did not provoke any significant changes in Caco-2 cells transcriptome when compared to the control condition (nIND).

**Figure 3:**
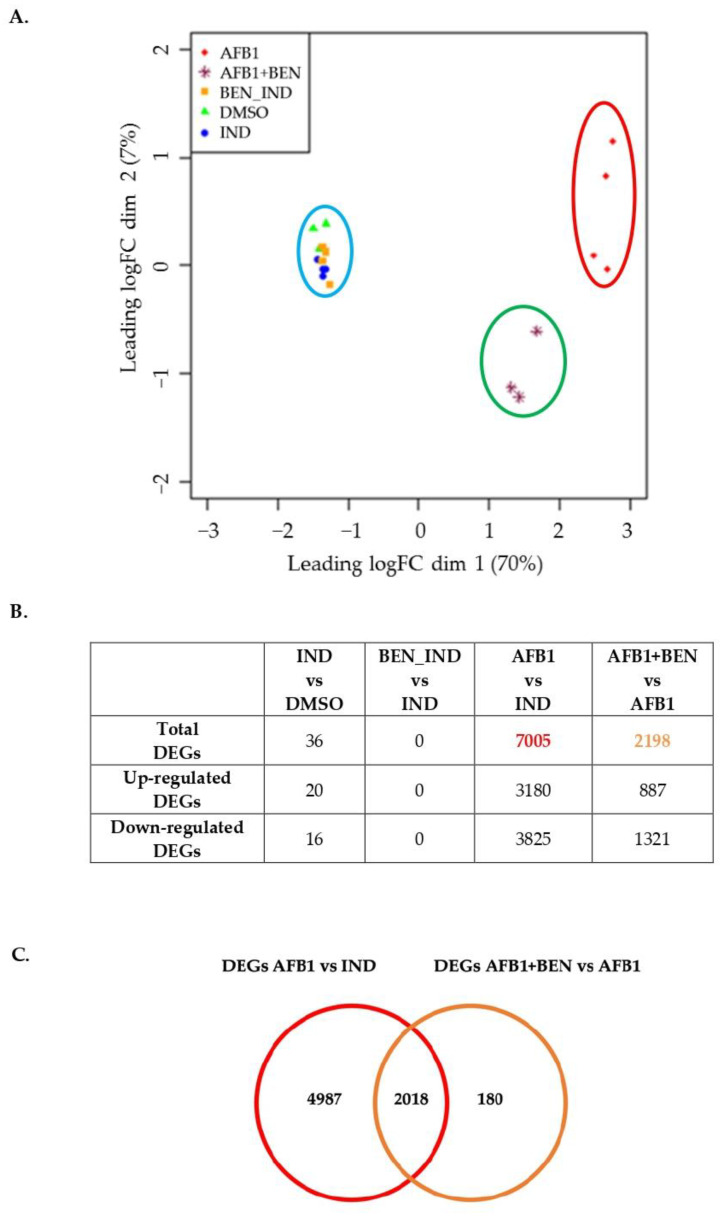
(**A**) MultiDimensional Scaling (MDS) plot of dataset including IND, BEN_IND, DMSO, AFB1 and AFB1 + BEN experimental groups. (**B**) Table reporting the significant DEGs obtained from the statistical analysis (FDR ≤ 0.05, and log fold change ≥ 1) of the following contrasts: IND vs. DMSO, BEN_IND vs. IND, AFB1 vs. IND and AFB1 + BEN vs. AFB1. (**C**) Venn diagram with the number of common DEGs between AFB1 vs. IND and AFB1 + BEN vs. AFB1.

As far as the analysis focusing on DMSO, IND, BEN_IND, AFB1 and AFB1 + BEN experimental groups is concerned, significant results were obtained. The MDS Plot gave the first hint about data similarities and differences in gene expression, giving a two-dimension synthetic graphical representation (Figure 3A).

As shown in Figure 3A, the first dimension (*x* axis) explained most of the observed variability (i.e., 70%) and divided samples into three main groups. Aflatoxin B1 samples (i.e., red circle, Figure 3A) clustered far away from IND, DMSO and BEN_IND samples (i.e., blue circle) which, in turn, were closed to each other; interestingly, AFB1 + BEN samples (i.e., green circle) clustered in between of the two aforementioned clusters. The number of DEGs detected by the QLF-test are reported in Figure 3B; the complete output is reported in Table S3.

Bentonite did not cause significant changes in gene expression even in the presence of the induction pre-treatment. The analyses of AFB1 vs. IND and AFB1 + BEN vs. AFB1 highlighted a great number of DEGs; shared DEGs were identified, too. A total of 2018 DEGs in AFB1 vs IND appeared to be differentially expressed also in AFB1 + BEN vs. AFB1 (Figure 3C). It is noteworthy that 2008 out of these 2018 DEGs showed an opposite behavior in the two experimental conditions; this would confirm the protective role of the clay against the toxic effect induced by AFB1 also at the transcriptomic level.

#### 2.5.2. Functional Enrichment and Gene Set Enrichment Analyses (GSEA)

The complete outputs of Gene Ontology (GO), Kyoto Encyclopedia of Genes and Genomes (KEGG) enrichment analyses and GO GSEA for the comparisons considered in the following lines were reported in the Supplementary Material section, in particular in Tables S4–S6, respectively.

According to GO enrichment analysis, when genes significantly up-regulated by AFB1 were compared with the control (IND), 11 biological processes were enriched; however, 136 biological processes enriched by genes were significantly down-regulated. Additionally, a total of 15 pathways were identified by the KEGG over-representation test performed on both up- and down-regulated DEGs.

**Figure 4:**
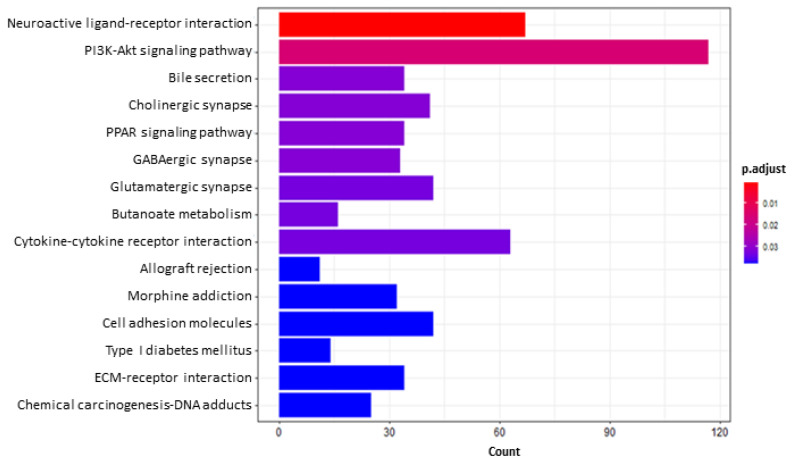
Bar plot of KEGG enrichment analysis (all DEGs) of aflatoxin B1 (AFB1) vs. control induced (IND) cells. The *X* axis reports the DEGs representing each pathway. The color gradient corresponds to the level of significance, adjusted with the Benjamini–Hochberg method.

Some GO terms identified among genes down-regulated by AFB1 are worth mentioning; in particular, those linked to “assembly” and “organization of cell junctions” (GO:0034329, 88 genes; GO:0034330, 152 genes, respectively). Within these ones, some interesting DEGs were noticed: for example, claudins 16 (*CLDN16*; log fold change, AFB1 vs IND, LFC_AvsI_ = −2.69) and 19 (*CLDN19*; LFC_AvsI_ = −1.95); the tight junction protein 2 (*TJP2*; LFC_AvsI_ = −1.50); the proto-oncogene tyrosine-protein kinase Src (*SRC*; LFC_AvsI_ = −1.75), and the protein tyrosine kinase 2 (*PTK2*; LFC_AvsI_ = −2.20). Accordingly, the KEGG pathway “cell adhesion molecules” (hsa04514, 42 genes) was over-represented (Figure 4). Further down-regulated GO terms are the “response to a xenobiotic stimulus”, “a toxic substance” or “to wounding” (GO:0009410, 112 genes; GO:0071466, 46 genes; GO:0009636, 62 genes; GO:0009611, 119 genes). These GO terms include transcripts linked to xenobiotic metabolism and transport, i.e., catalase (*CAT*; LFC_AvsI_ = −1.71), *CYP2W1* (LFC_AvsI_ = −1.75), the ATP binding cassette (ABC) subfamily A member 1 (*ABCA1*; LFC_AvsI_ = −3.63), *ABCC1* (LFC_AvsI_ = −1.50) and *ABCC2* (LFC_AvsI_ = −1.53). Interestingly, with the exception of *ABCC1* and *ABCC2*, BEN significantly protected cells from AFB1-dependent gene down-regulation.

Aflatoxin B1 is known to be carcinogenic, and in our experimental conditions, the Wnt signaling pathway (GO:0030111, 79 genes), known to play a significant role in carcinogenesis, was repressed; however, the KEGG pathway “chemical carcinogenesis-DNA adducts” (hsa05204, 25 genes) is enriched in the presence of AFB1.

In addition, AFB1 affected some GO terms linked to cellular basal metabolism; namely, those involved in “cholesterol homeostasis” and “lipid catabolic process”, “localization” and “transport” (GO:0042632, 28 genes; GO:0016042, 84 genes; GO:0046486, 106 genes; GO:0010876, 110 genes; GO:0006869, 100 genes) as well as in “insulin receptor signaling pathway”, “insulin secretion” and “response” (GO:0008286, 40 genes; GO:0030073, 51 genes; GO:0032868, 75 genes). Among the DEGs, we can mention the insulin-like growth factor 1 receptor (*IGF1R*; LFC_AvsI_ = −3.44), the insulin receptor (*INSR*; LFC_AvsI_ = −2.50), and the insulin-like growth factor 2 (*IGF-2*; LFC_AvsI_ = −1.11); the glycogen synthase kinase 3 beta (*GSK3B*; LFC_AvsI_ = −1.61); the phosphoinositide-3-kinase regulatory subunit 1 (*PIK3R1*; LFC_AvsI_ = −1.06), the phosphatidylinositol-4,5-bisphosphate 3-kinase catalytic subunit beta (*PIK3CB*, LFC_AvsI_ = −1.96), the phosphoinositide-3-kinase adaptor protein 1 (*PIK3AP1*, LFC_AvsI_ = −2.12); the mechanistic target of rapamycin kinase (*m-TOR*; LFC_AvsI_ = −1.01), the forkhead box O1 (*FOXO1*; LFC_AvsI_= −1.04) and *FOXO4* (LFC_AvsI_ = −1.09); the peroxisome proliferator activated receptor gamma (*PPAR-γ*; LFC_AvsI_= −1.09) and *PPAR-α* (LFC_AvsI_ = −1.01). These data are supported by KEGG pathways such as “PI3K-Akt signaling pathway” (hsa04151, 117 genes), “PPAR signaling pathway” (hsa03320, 34 genes) and “type I diabetes mellitus” (hsa04940, 14 genes). Finally, AFB1 negatively influenced the cellular ion balance, as shown by the involvement of GO terms linked to ion transport and homeostasis (GO:0006813, 41 genes; GO:0006814, 69 genes; GO:0006873, 126 genes; GO:0034765, 91 genes; GO:0035725, 48 genes; GO:0043269, 133 genes; GO:0071805, 40 genes).

**Figure 5:**
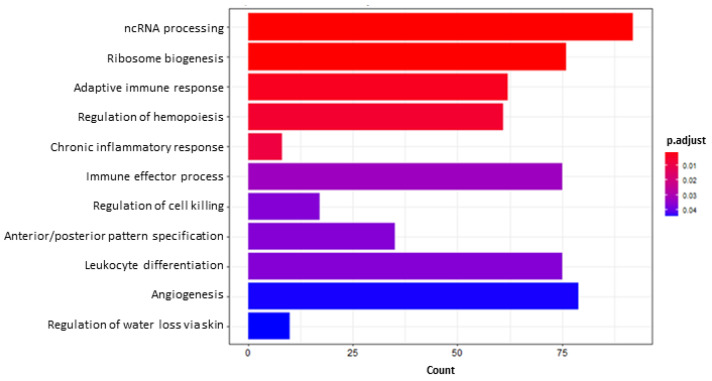
Bar plot of GO enrichment analysis of aflatoxin B1 (AFB1) vs. control induced (IND) up-regulated genes. The *X* axis reports the number of DEGs representing each pathway. The color gradient corresponds to the level of significance that is adjusted with the false discovery rate method.

Conversely, the GO terms enriched when analyzing up-regulated DEGs are related to immune and inflammatory response (e.g., GO:0002544, 8 genes; GO:0002252, 75 genes; GO:0031341, 17 genes; GO:0002521, 75 genes; GO:0002250, 62 genes) as well as angiogenesis (GO:0001525, 79 genes; Figure 5). Concerning the first one, we noticed the up-regulation of tumor necrosis factor (*TNF*; LFC_AvsI_ = 5.61), toll-like receptor 4 (*TLR4*; LFC_AvsI_ = 1.61), interleukin 11 (*IL11*; LFC_AvsI_ = 2.71), *IL5* (LFC_AvsI_ = 3.45) and the aryl hydrocarbon receptor (*AHR*; LFC_AvsI_ = 1.25); within the second one, the growth arrest and DNA damage inducible alpha (*GADD45A*; LFC_AvsI_ = 1.18). Further up-regulated DEGs are of interest; specifically, those involved in the oxidative stress response such the glutathione peroxidase 1 (*GPX1*; LFC_AvsI_ = 1.44) and *GPX2* (LFC_AvsI_ = 2.40).

The GO GSEA allowed us to confirm some of the abovementioned pathways. A total of 268 GO terms were enriched. Of particular interest are those resulted significant also in GO and KEGG over-representation analysis, such as those ones linked to “cell junction organization” and “assembly” (GO:0034330, 485 genes; GO:0034329, 287 genes; GO:1901888, 135 genes) or to “response to insulin” (GO:0032868, 217 genes). Additional GO terms providing the reader a more complete picture refer to intrinsic and extrinsic apoptosis (GO:0042771, 40 genes; GO:1902042, 24 genes; GO:2001242, 145 genes), “negative regulation of TORC1 signaling” (GO:1904262, 15 genes) and “regulation of response to DNA damage stimulus” (GO:2001020, 206 genes).

When comparing the enriched GO terms obtainable with AFB1 vs. IND and AFB1 + BEN vs. AFB1 comparisons, we observed that AFB1 inhibits pathways linked to digestion, absorption and metabolism of macronutrients; as an example, “lipid transport” (GO:0006869), “lipid catabolic process” (GO:0016042) and “intestinal absorption” (GO:0050892); however, and worthy of mention, an opposite behavior was noticed after AFB1 and BEN co-exposure. In addition, the “sulfur compound metabolic process” (GO:0006790) showed a similar trend; among DEGs involved in AFB1 mechanistic toxicology we found glutathione S-transferase alpha 1 (*GSTA1*; LFC_AvsI_ = −3.06, LFC, AFB1 + BEN vs AFB1, LFC_ABvsA_ = 1.74), *GSTA2* (LFC_AvsI_ = −1.69, LFC_ABvsA_ = 1.10) and *GSTM4* (LFC_AvsI_ = −2.28, LFC_ABvsA_ = 1.45).

The functional analysis of DEGs resulting from the comparison between AFB1 + BEN vs. AFB1 highlighted 37 and 136 up- and down-regulated GO classes, respectively; moreover, 10 KEGG pathways and 157 GO GSEA terms were enriched, too. It is worth mentioning that the AFB1 + BEN co-exposure down-regulated not only some GO terms up-regulated by AFB1, e.g., “adaptive immune response” or “immune effector process”(GO:0002250, 23 genes; GO:0002252, 29 genes) and “angiogenesis” (GO:0001525, 31 genes), but also “DNA packaging”, “nucleosome assembly” and “organization” (GO:0006323, 20 genes; GO:0006334, 18 genes; GO:0034728, 19 genes). Among the over-represented KEGG pathways, the “Rap1 signaling pathway” (hsa04015, 30 genes), involved in cell adhesion and junctions formation, was found enriched.

**Figure 6:**
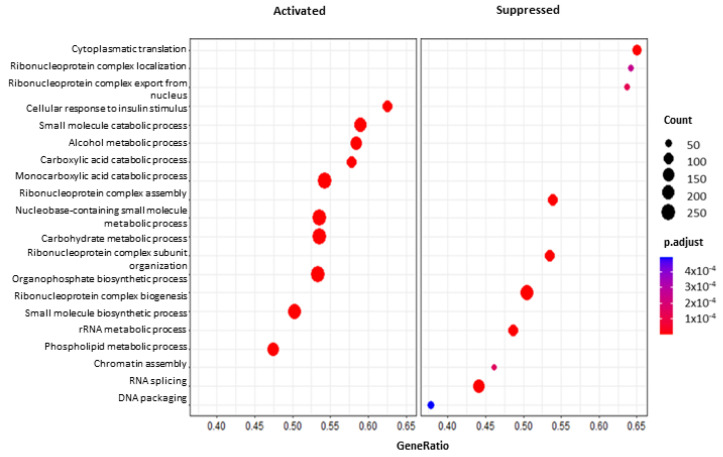
Dot plot of the 20 most significant pathways as results of GO GSEA of AFB1 + BEN vs. AFB1. Dot size represents the number of genes belonging to each pathway. The color gradient is related to the level of significance, adjusted with the Benjamini–Hochberg method. The box on the left collects activated pathways, while the box on the right the suppressed ones.

Concerning the GO GSEA (Figure 6), among the positively enriched pathways, we noticed post-translation modifications such as “glycosylation” and “dephosphorylation” (GO:0070085, 196 genes; GO:0006486, 185 genes; GO:0043413, 185 genes; GO:0016311, 346 genes; GO:0006470, 232 genes) and also “cellular response to insulin stimulus”, “positive regulation of glucose import”, “carbohydrate homeostasis”, “transmembrane transport” and “metabolic process”(GO:0032869, 171 genes; GO:0046326, 29 genes; GO:0033500, 188 genes; GO:0034219, 91 genes; GO:0005975, 480 genes). Even “NAD” and “NADH metabolic process” (GO:0019674, 24 genes; GO:0006734, 24 genes) were found up-regulated.

**Figure 7:**
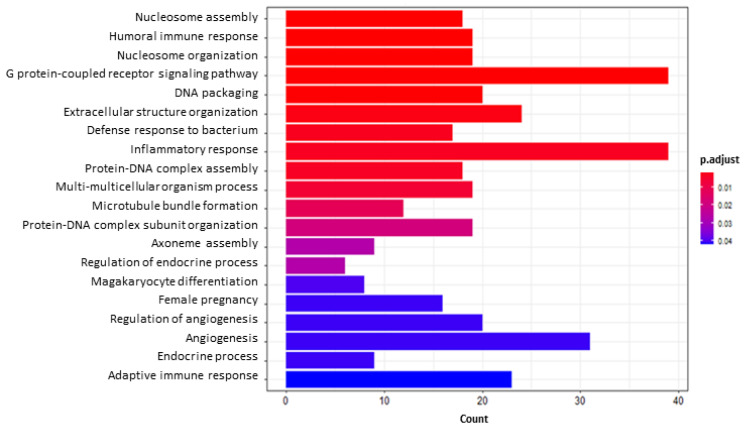
Bar plot of the 20 most significant pathways as results of GO enrichment analysis of aflatoxin B1 (AFB1) + bentonite (BEN) vs. AFB1 down-regulated genes. *X* axis reports the number of genes representing each pathway. The color gradient corresponds to the level of significance that is adjusted with the false discovery rate method.

Additionally (Figure 7), the co-treatment negatively regulated terms linked to “DNA packaging” and “chromatin assembly” (GO:0006323, 127 genes; GO:0031497, 91 genes), “mRNA transport”, ”RNA splicing” and “transcription by RNA-polymerase I” (GO:0051028, 117 genes; GO:0008380, 372 genes; GO:0006360, 52 genes). Moreover, it is interesting to remember “ribonucleoprotein complex localization”, “export from nucleus”, “organization”, “biogenesis” and “assembly” (GO:0071166, 70 genes; GO:0071426, 69 genes; GO:0071826, 189 genes; GO:0022613, 418 genes; GO:0022618, 182 genes) and “DNA damage response, signal transduction by p53 class mediator resulting in cell cycle arrest” (GO:0006977, 17 genes) were found negatively enriched, too.

Interestingly, the GO GSEA analysis of AFB1 vs. IND highlighted two pathways correlated to a previous one, i.e., the “intrinsic apoptotic signaling pathway in response to DNA damage by p53 class mediator” (GO:0042771, 40 genes) and the “positive regulation of signal transduction by p53 class mediator” (GO:1901798, 23 genes). Indeed, the tumor protein p53 (*TP53*; LFC_AvsI_ = 1.63, LFC_ABvsA_ = −0.72), the p53-induced death domain protein 1 (*PIDD1*; LFC_AvsI_ = 2.51, LFC_ABvsA_ = −1.33), and the phorbol-12-myristate-13-acetate-induced protein 1 (*PMAIP1*; LFC_AvsI_ = 6.16, LFC_ABvsA_ = −2.46) were down-regulated in cells co-treated with AFB1 and BEN; on the contrary, they were up-regulated by AFB1. It is worth noting that even transcription of B-cell lymphoma 2-like 14 (*BCL2-L14*; LFC_AvsI_ = −2.17, LFC_ABvsA_ = 1.27) and proliferating cell nuclear antigen (*PCNA*; LFC_AvsI_ = 1.12, LFC_ABvsA_ = −0.24) were influenced by AFB1, thus supporting the role of this mycotoxin in the regulation of apoptosis.

**Figure 8:**
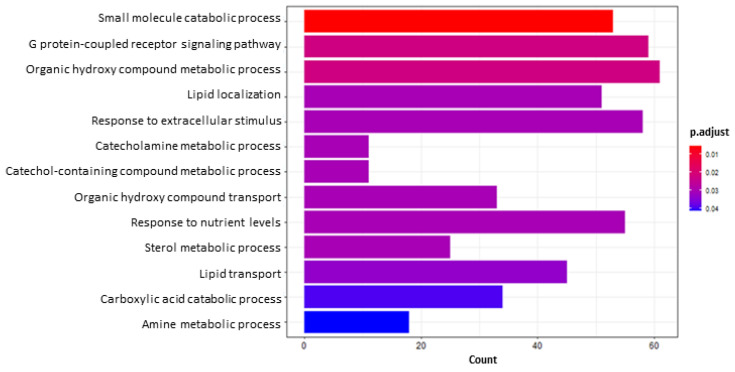
Bar plot GO enrichment analysis of DEGs in common between AFB1 vs. IND and AFB1 + BEN vs. AFB1. *X* axis reports the number of genes representing each pathway. The color gradient corresponds to the level of significance that is adjusted with the false discovery rate method.

Looking in-depth into DEGs and pathways ideally considered as BEN responsive (when it is used as a reliever of AFB1 toxicity), a specific search on the 2018 DEGs shared between AFB1 vs. IND and AFB1 + BEN vs. AFB1 was performed. These “selective” approaches led to the enrichment of 13 GO terms (Figure 8), particularly the “response to extracellular stimulus” (GO:0009991, 58 genes).

**Figure 9:**
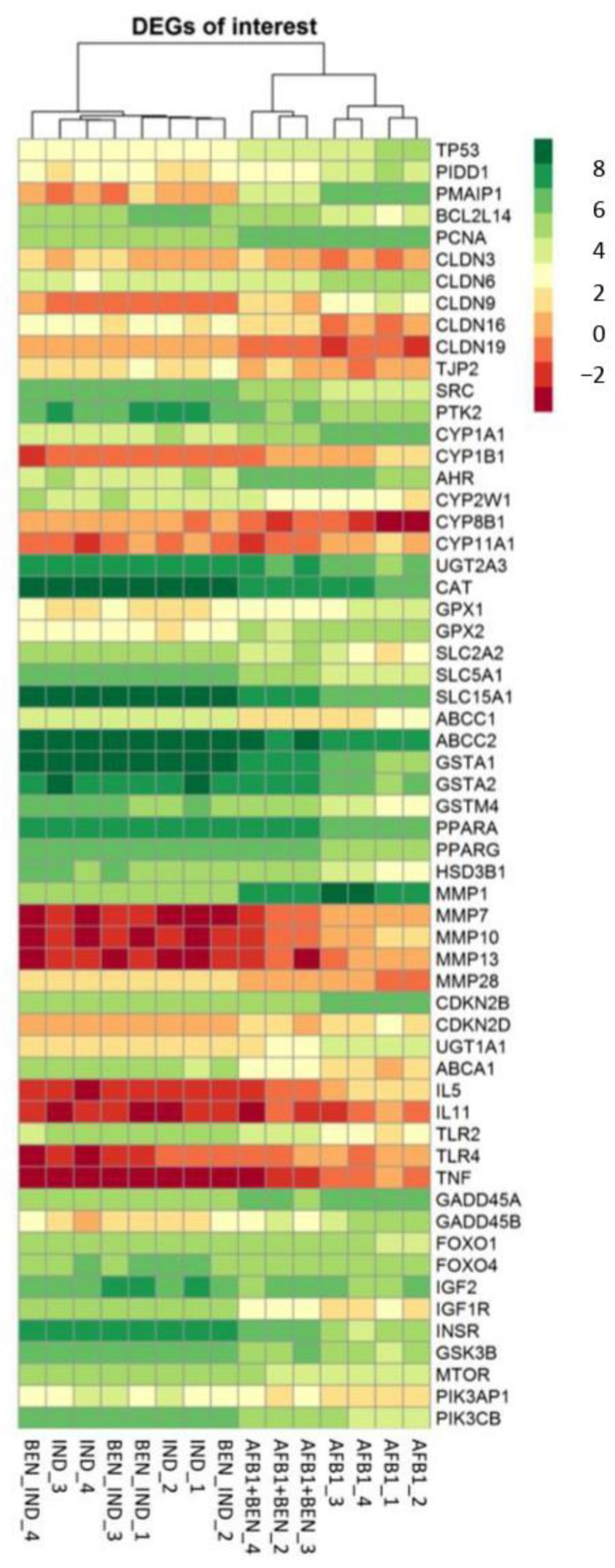
Heatmap of DEGs considered of interest according to pathways highlighted in the functional analysis, in induced (IND) cells as well as in IND cells exposed to bentonite (BEN_IND), aflatoxin B1 (AFB1) and the co-treatment (AFB1 + BEN). Data are expressed as log counts per million (logCPM).

Notably, the metalloproteinase 7 (*MMP7*; LFC_AvsI_ = 4.13, LFC_ABvsA_ = −1.47), the UDP glucuronosyltransferase family 1 member A1 (*UGT1A1*; LFC_AvsI_ = 2.67, LFC_ABvsA_ = −1.72), *ABCA1* (LFC_AvsI_ = −3.63, LFC_ABvsA_ = 1.55), *CYP8B1* (LFC_AvsI_= −2.98, LFC_ABvsA_= 1.68), *CYP26B1* (LFC_AvsI_ = 1.98, LFC_ABvsA_= −1.44), the cyclin dependent kinase inhibitor 2D (*CDKN2D*; LFC_AvsI_ =2.12, LFC_ABvsA_ = −1.27) and *CDKN2B* (LFC_AvsI_ = 1.23, LFC_ABvsA_ = −1.15) belonged to this pathway. We also found DEGs involved in different pathways; for example, in the immune response, e.g., *TLR2* (LFC_AvsI_ = −2.50, LFC_ABvsA_ = 1.47); cells junctions and invasion, i.e., *MMP10* (LFC_AvsI_ = 4.01, LFC_ABvsA_ = −2.01 and *MMP28* (LFC_AvsI_ = −2.25, LFC_ABvsA_ = 1.06), *CLDN6* (LFC_AvsI_ = 1.32, LFC_ABvsA_ = −1.11) and *CLDN9* (LFC_AvsI_ = 4.24, LFC_ABvsA_ = −2.14). It is worth noting that *MMP1* (LFC_AvsI_ = 2.79, LFC_ABvsA_ = −0.93) and *CLDN3* (LFC_AvsI_ = −0.94, LFC_ABvsA_ = 0.91) showed a significant difference in gene expression, but the LFC value is slightly below our cutoff for one or both comparisons, respectively. Within genes coding for enzymes involved in metabolism and detoxification, we could mention the hydroxy-delta-5-steroid dehydrogenase, the 3-beta- and steroid delta-isomerase 1 (*HSD3B1*; LFC_AvsI_ = −1.90, LFC_ABvsA_= 1.38), *UGT2A3* (LFC_AvsI_ = −1.77, LFC_ABvsA_ = 1.02), *CYP11A1* (LFC_AvsI_ = 1.15, LFC_ABvsA_ = −1.96), *CYP1B1* (LFC_AvsI_ = 1.86, LFC_ABvsA_ = −1.19) and *CYP1A1* (LFC_AvsI_ = 1.92, LFC_ABvsA_ = −0.82), although the LFC value of this latter one was slightly under our cutoff in AFB1 + BEN vs. AFB1. A number of the solute carrier (SLC) superfamily of transporters showed significant differences in gene expression, namely *SLC15A1* (LFC_AvsI_ = −2.51, LFC_ABvsA_ = 1.01), *SLC2A2* (LFC_AvsI_ = −2.54, LFC_ABvsA_ = 1.59) and *SLC5A1* (LFC_AvsI_ = −2.50, LFC_ABvsA_ = 1.06). The heatmap showing the level of expression of the DEGs of major interest in the four experimental groups (IND, BEN, AFB1 and AFB1 + BEN) is reported in Figure 9.

## 3. Discussion

As a whole, the present study confirms the ability of BEN to decrease AFB1 toxicity thanks to its adsorbent properties; moreover, it suggests this clay may affect the viability of Caco-2 cells without altering the monolayer integrity.

The bentonite clay is reported by EFSA as a safe substance for all animal species when used as an additive to bind mycotoxins in animal feed at a maximum level of 20,000 mg/kg [24]. Moreover, it is effective in reducing the toxicity caused by contaminated feed in vivo in poultry, pigs and dairy cows [35,36,37,38]. Numerous in vitro studies have investigated the binding properties and efficacy of BEN as a detoxifying agent; however, little is known about the effect of this clay on the gastrointestinal cells at the transcriptomic level [39,40].

### 3.1. 12-O-Tetradecanoylphorbol 13-Acetate and NaB-Mediated Induction of CYP3A4

Caco-2 cells are derived from a human colorectal adenocarcinoma and represent a well-known model for permeability and adsorption studies since they spontaneously differentiate into polarized cells with high morphological and physiological similarities with enterocytes of the human small intestine [31,34,41,42,43]. These cells have already been used to evaluate the effect of BEN in preventing mycotoxins toxicity [21,44,45]. Although they represent a valid gastrointestinal tract in vitro model, Caco-2 cells present some limitations when it comes to drug metabolism studies; in fact, they lack CYP3A4, which is the principal CYP isoform found in the human intestine and responsible for the metabolism of more than 50% of drugs and also the one primarily involved in the bioactivation of AFB1 in humans [46,47,48,49,50,51]. To overcome this limitation, various strategies have been used. The most common approach is to enhance CYP3A4-mediated metabolism (1) by transfecting Caco-2 cells with cDNA encoding for CYP3A4 or transcription factors that are natural activators of CYP3A4; or (2) by exposing the cells to 1α,25-dihydroxyvitamin D3 or (3) TPA and NaB [46,47,52,53,54]. In our study, we followed the protocol of Cummins et al. because of its low cost and ease of use, and we used fully differentiated Caco-2 cells [52]. The treatment with NaB and TPA resulted in a significant increase, albeit of modest magnitude, in *CYP3A4* mRNA expression, and significantly higher cytotoxicity to AFB1 when the highest AFB1 concentration was tested (90 µM). Furthermore, this slight induction allowed us to better appreciate the beneficial effect of BEN in decreasing AFB1 toxicity in the co-incubation studies.

### 3.2. Analytical Investigations (LC-MS/MS)

It is an established concept that BEN and other clays adsorb AFB1, and in vitro, this interaction occurs in extracellular medium or in solutions mimicking the gastrointestinal tract chemical–physical characteristics (e.g., pH) [21,55,56]. Our results would confirm that BEN binds AFB1 in the medium. This hypothesis is also supported by our analytical investigations, which confirmed the ability of BEN to adsorb the mycotoxin and its derivatives. In this respect, data concerning AFL seem to be of particular interest. In our experimental conditions, AFL was adsorbed to a greater extent (~50.0%) compared to AFB1 and AFM1. The reduction of AFB1 to AFL (~10%) and the reconversion (oxidation) of AFL to AFB1 occur in many species, and AFL has been proposed as a reservoir of AFB1 in sensitive species (e.g., duck, trout); however, the pattern of AFB1 reduction to AFL in human liver subcellular fractions is still controversial, as it varies from scarce to moderate [57,58]; therefore, although we proved that AFB1 is converted to AFL in IND Caco-2 cells, and BEN may reduce by ~50% the total amount of free AFL, more research is needed to better determine the kinetics of AFL (e.g., absorption and metabolism), and to understand whether BEN adsorption of AFL might substantially contribute to lower AFB1 toxicity. It is worth noting that no data about the adsorbing properties of BEN toward AFL are actually available.

### 3.3. Cytotoxicity, Permeability and Trans-Epithelial Electric Resistance Evaluation

It is commonly believed that clays are not cytotoxic, and BEN does not affect Caco-2 cell viability [21]; however, a fair number of publications suggest that clays are toxic in vitro, with some differences attributable to the type of clay, concentration and time of exposure [26,44,45,59,60,61,62,63]. As to BEN, it induces necrosis and apoptosis in the IMR90 cell line and HMy2.CIR cell line; furthermore, a modified form of montmorillonite is genotoxic in the Caco-2 cell line [45,60,61]. In our experimental conditions, BEN is cytotoxic to Caco-2 cells, with similar IC_50_ values in IND and nIND cells (0.08 mg/mL and 0.09 mg/mL, respectively). Hence, we speculate that CYP3A4 does not play a major role in BEN cytotoxicity. Interestingly, similar behavior has never been reported so far. Concerning the protective role of BEN against AFB1 cytotoxicity, a bimodal dose-dependent behavior was observed. Up to 0.1 mg/mL (the most effective concentration), BEN was successful in preventing AFB1 cytotoxicity, as expected. By contrast, an opposite behavior was observed with higher BEN concentrations (0.6 and 1.2 mg/mL). It is worth noting that the most effective BEN concentration (i.e., the one chosen for the remaining part of the study) is quite close to IC_50_ values calculated for BEN alone. To make the scenario of results more complex, BEN at 0.1 mg/mL did not alter the Caco-2 monolayer integrity, and protected against AFB1-dependent increased permeability (although not significantly). As we confirmed, also by analytical investigations, that BEN adsorbs AFB1 (and its derivatives) in the cell medium, we may hypothesize that in the absence of AFB1 the clay binds essential components in the media, thus leading to a decreased cell viability [64]. Taking all these pieces of evidence as a whole, we suggest that further studies are needed not only to support our hypotheses, but also to better clarify the possible mechanisms involved in the observed BEN dose-dependent cytotoxicity, the minor effects on cell permeability and its protective (adsorbent) effects toward AFB1 and its derivatives (mostly, AFL).

### 3.4. Whole-Transcriptomic (RNA-seq) Investigations

Taking as a starting point both the abovementioned results and the resulting perspectives, we investigated the transcriptional effects of BEN, used either alone, at the most effective concentration (0.1 mg/mL), or in combination with AFB1 (co-exposure), on Caco-2 cells. To pursue this goal, an RNA-seq study was performed. It is worth mentioning that no data about the whole-transcriptomic effects of BEN, assessed by using this –omic technique, have been published so far. The overall approach to discuss our results was to consider the most relevant transcriptional effects of AFB1 on Caco-2 cells for which BEN was preventive, improving or not affecting the overall mycotoxin toxicity.

The first comparison aimed at discovering the transcriptional changes induced by BEN 0.1 mg/mL on both IND and nIND cell monolayers. Data obtained showed the clay did not cause significant perturbations in the Caco-2 cell transcriptome. In this regard, it should be emphasized that the BEN concentration suggested by manufacturers is usually based on AFB1:clay ratio calculation. As said before, bimodal BEN dose-dependent cytotoxic effects have been observed; moreover, BEN 0.1 mg/mL was the highest and the most effective BEN concentration, but higher concentrations were proved cytotoxic; therefore, caution must be given when affirming that BEN in basal conditions is safe and does not provoke significant changes in cell transcriptome, independently from the used concentration. Conversely, AFB1 showed a very strong impact on the Caco-2 cell transcriptome. Furthermore, when looking at and comparing the AFB1 and the co-exposure results, a high percentage of common DEGs was observed; hence, the greatest contribution to the observed transcriptional changes is attributable to AFB1 and not to BEN. Hence, to better describe and explain RNA-seq results, it is more appropriate to start from the data more easily linked to the aforementioned empirical observations.

As far as AFB1 effects on enterocyte monolayers, some tight junction proteins (e.g., *TJP2* and claudins) were compromised, in accordance with recent studies describing mycotoxin-related alterations of the intestinal barrier [65,66]. In particular, *CLDN3*, *16*, and *19* were down-regulated, while *CLDN6* and *9* were up-regulated. The first three (*CLDN3*, *6* and *19*) belong to the pore-sealing group of claudins [66,67]; moreover, a down-regulation of the *CLDN3* gene was noticed in former studies in which Caco-2 cells were exposed to lower AFB1 concentrations [44,68]; finally, broilers and *Sparus aurata* exposed to AFB1 in vivo showed similar alterations in a number of *zonula occludens* members (i.e., tight-junction proteins) [69,70]. Focusing on adhesion molecules, it is important to highlight that two tyrosine kinases, namely *SRC* and *PTK2* (fak), were down-regulated by AFB1. By contrast, the co-exposure with BEN reduced the amplitude of such a down-regulation, albeit slightly below our selected cut-off value. Proteins coded by these two genes are physically and functionally related to each other and also linked to the insulin-*like growth factor* receptor 1 (*IGFR1*) activity; they play a relevant role in cancer migration and progression as well as in modulating cellular adhesions, proliferation and interactions with extracellular matrix [71,72].

Apart from the possible effects of AFB1 on cell permeability and integrity, a focus on xenobiotic-metabolizing enzymes is of interest [73] since the gastrointestinal tract is one of the major sites of extrahepatic metabolism. As to oxidative (phase I) xenobiotic metabolism, *CYP1A1* is known to participate in the bioactivation of AFB1 in its epoxide metabolite; moreover, it is the foremost extra-hepatic member of the CYP1A subfamily. Both *CYP1A1* and *CYP1B1* are regulated by *AHR*, which was also up-regulated after AFB1 exposure. Though the AFB1 planar structure could suggest it may act as an *AHR* activator, our information does not allow us to hypothesize an AFB1-dependent induction of this nuclear receptor [74]. Other members of the CYP family appeared to be influenced by both AFB1 and BEN co-treatment; for example, *CYP2U1* and *CYP2W1*, two genes highly expressed in colorectal cancer [75,76,77]—*CYP11A1*, involved in steroidogenesis and down-regulated by zearalenone in porcine Leydig cells [78]; *CYP8B1*, a gene coding for a key enzyme in bile acids synthesis [79]. Among the conjugative (phase II) xenobiotic-metabolizing enzymes, we include UGTs and GSTs, which are responsible for the xenobiotic (the parent compound or its derivatives) conjugation with glucuronic acid and glutathione (GSH), respectively. As to UGTs, the *UGT1A1* gene was induced by AFB1, while *UGT2A3* was inhibited. All *UGT1As* are up-regulated by AFB1 in HepG2 cells [80]. The AFB1 detoxification reactions, apart from epoxide hydrolase, seem to also involve cytosolic GSTs, such as *GSTA1*, *GSTA2* and *GSTM4*, which allow the conjugation of epoxide metabolite with GSH [11]. The general down-regulation of these three genes by AFB1 seemed to be partially counteracted by the co-treatment with BEN. Interestingly, lower *GSTA1* and *GSTA2* mRNA levels were observed in BME-UV1 cells exposed to AFB1 [81].

Antioxidant enzymes, and particularly GPXs, are a hinge of AFB1 detoxification pathways [82], and flavonoids and curcuminoids (possessing antioxidant derivatives) have recently been shown to mitigate AFB1 toxic effects in vitro [83,84]. By contrast, in our experimental conditions, BEN did not show similar behavior. Both *GPX1* and *2* were up-regulated by AFB1, while the co-exposure down-regulated them (i.e., below 1.5 fold change, FC). Glutathione peroxidases defend cells from oxidative stress; in particular, breaking down hydrogen peroxides and inhibiting lipid peroxidation. The most abundant and ubiquitous GPX gene is *GPX1*, while *GPX2* is mostly expressed in the gastrointestinal tract [85]. Interestingly, *GPX1* function correlates also with that of *p53*, a known tumor-suppressor gene and an anti-apoptotic element [86]. Among the triad of genes coding for antioxidant enzymes (superoxide dismutase, *CAT* and *GPX*), *CAT* expression was also influenced by AFB1. This gene is down-regulated, in agreement with other authors who described a reduced catalase activity after AF exposure on mice brain, piglet mesenteric lymph nodes and chicken duodenal tissue [87,88,89].

Another way by which the intestinal barrier reacts to xenobiotics, including mycotoxins, is their active efflux through ABC-transporters [90,91,92]. Overall, in our study, some efflux ABC-transporters showed an altered gene expression pattern. Specifically, AFB1 seems to down-regulate *ABCC2* and *ABCC1* (mrp1), with BEN that only partially (below the FC cutoff value) reduced the impact of such AFB1-dependent decrease in their mRNA levels. Present results disagree with those obtained by Huskoneen and colleagues, that observed increasing mRNA levels of *ABCC2* in the trophoblastic JEG-3 cell line exposed to AFB1 (2–6 µM) [93]. We claimed that the potential discrepancies between our findings and the literature could be related to the different cell lines used, the chosen AFB1 concentrations and, last but not least, possible kinetic AFB1-carrier-specific relationships (e.g., affinity, carrier saturation and competitive inhibition) [3,92,94]. Within the large class of transporters whose expression is affected by mycotoxins, we also include the SLC superfamily of afflux transporters [95,96]. Given the effect of AFB1 on mRNA levels of these transporters, we suppose that the mycotoxin negatively influenced steroids, peptides and glucose transport and metabolism [97,98,99]. For example, we observed the down-regulation of *ABCA1*, *SLC15A1* (pept1), *SLC2A2* (glut2) and *SLC5A1* (sglt1) genes; on the other hand, a restoration of their mRNA levels was noticed in the presence of BEN.

Taking into consideration the effects of AFB1 on *SLC2A2* and *SLC5A1*, which are the main regulators of intestinal glucose absorption and efflux [97], we looked at transcripts involved in the insulin response. We observed an AFB1-mediated inhibition of genes such as *IGF1R*, *INSR*, *IGF-2*, *GSK3B*, *PIK3R1*, *PI3KCB*, *PIK3AP1*, *FOXO1*, *FOXO4* and the mechanistic target of rapamycin (*mTOR*). When using lower AFB1 concentrations and different cell lines, *IGFR1* and *IGF-2* were up-regulated [100]; however, a recent study that correlated the exposure to AFM1 and the presence of metabolic disorders in a human population, including diabetes mellitus [101], corroborates our findings. As to *FOXO* genes, they are transcription factors related to insulin and igf-1 activity; moreover, they are involved in several pathways linked to metabolism and oxidative stress. We hypothesize that the down-regulation of some players involved in the insulin response pathway could cause the consequent inhibition of these transcription factors [102]. Additionally, *mTOR* is a kinase within the mTOR complex 1 (*mTORC1*), which plays a key role in regulating cellular response to nutrients and growth factors such as insulin, including the intervention of the PI3K/Akt pathway. Moreover, it seems to have a role in the activation and inhibition of *PPAR-γ* and *PPAR-α*, respectively. As a whole, and primarily considering the overall trend to gene down-regulation, we hypothesize that AFB1 exposure provokes a dysregulation of glucose and lipid metabolism [103]. It is worth noting that *INSR*, *PI3KCB* and *PIK3AP1* genes showed an opposite trend of expression (no down-regulation) following cells’ co-exposure with BEN.

Apart from glucose homeostasis, also steroids metabolism appears to be affected by AFB1. Aside from the abovementioned *CYP11A1*, *ABCA1* and *ABCG4*, *HSD3B1* and *UGTA1* were also modulated by AFB1. In particular, these two genes were down-regulated in AFB1 vs. IND and up-regulated in AFB1 + BEN vs. AFB1. Once again, our results disagree with those of Huskoneen et al.; indeed, these genes were up-regulated by AFB1 in JEG-3 cells; on the other hand, other mycotoxins (deoxynivalenol, zearalenone and T-2) decreased their mRNA levels in porcine Leydig cells [78,93].

A number of studies proved AFB1 causes inflammation; in addition, it shows immunomodulatory effects [89,104,105]. As a consequence, our attention was caught by the altered expression of *IL-5*, *IL-11*, *TNF*, *TLR-4* and *TLR-2*, and the protective effect shown by BEN. As to AFB1, an up-regulation of cytokine mRNA levels was observed in murine central-nervous-system-derived cells and splenocytes [106,107].

The p53 biological network is a key responder in cellular stress response. Apart from inflammation, p53 is involved in cell cycle arrest, apoptosis, DNA repair mechanisms and cell senescence. Aflatoxin B1 causes oxidative stress; furthermore, it indirectly induces mutations in codon 249 of TP53 by lipid peroxidation [108,109]. In our experimental conditions, AFB1 up-regulated *TP53* (p53) and *PIDD1*. Hence, we would confirm the involvement of AFB1 in cell cycle arrest, as hypothesized in former in vitro/in vivo experiments [110,111]. Interestingly, AFM1 was also considered as the causative agent of cell cycle arrest in differentiated Caco-2 cells [112]. Furthermore, within cell cycle arrest, it is worth mentioning the AFB1-dependent *PCNA* up-regulation. This gene seems to be involved in regulating genome stability; moreover, it is a possible target for *CDKN1A*, which induces a CDKs-independent cell cycle arrest in the S-phase [113]. Likewise, we underline the up-regulation of the other two members of the INK4 family of CDK inhibitors, i.e., *CDKN2B* (p15) and *CDKN2D* (p19). Interestingly, such an up-regulation was counterbalanced by BEN. These two DEGs control cell cycle progression through the G1 phase; moreover, *CDKN2D* also plays a key role in DNA repair under genotoxic stress conditions [114].

Moreover, in cell cycle arrest, *TP53* also participates in apoptosis. Nevertheless, our increasing *TP53* mRNA level is not the only clue about the possible involvement of AFB1 in programmed cell death. As a matter of fact, other DEGs encoding for members of the bcl-2 protein family, i.e., *BCL2-L14* and *PMAIP1* (noxa), were up-regulated in AFB1-exposed cells. Some authors observed a high constitutive expression of *BCL2-L14* in mice and human normal gastrointestinal tract; by contrast, this gene was down-regulated in inflammatory and tumor conditions. Overall, its biological role is still unclear. It seems at least partially involved in cellular protein transport and chemokine secretion, thus possessing a possible immunomodulatory role [115,116]. In our study, it was strongly down-regulated by AFB1, and partially restored in AFB1 + BEN vs. AFB1. Anyway, the frequent involvement of AFs in apoptosis has already been described in different cell lines, albeit with other techniques, e.g., flow cytometry [117,118]. An interesting outcome connecting apoptosis and cell cycle arrest is the up-regulation of two stress sensors, *GADD45A* and *B*. These genes are known to interact with p21 (*CDKN1A*), pcna, p38/jnk e atm/p53 [119,120].

When thinking about the carcinogenic nature of AFB1, a further and last point to be discussed is the expression of MMPs. They are zinc-dependent endopeptidases and their main function is to digest the extracellular matrix, being of pivotal relevance in carcinogenesis and metastatic progression [121]. In our study, we found an up-regulation of *MMP1*, *MMP7* and *MMP10* in AFB1-exposed cells, while a down-regulation was observed in AFB1 + BEN vs. AFB1. On the contrary, *MMP28* showed an opposite pattern of expression. Our results suggest AF may affect MMPs gene expression, as already shown in a former study [89], even though other MMP isoforms were taken into account.

## 4. Conclusions

To the best of our knowledge, this is the first study assessing the in vitro toxicity and whole-transcriptomic effects of BEN, one of the most commonly used clay to adsorb AFB1. For this purpose, Caco-2 cells were exposed to BEN alone or in combination with AFB1. As a whole, our data suggest that: (1) The clay binds AFB1, AFM1 and AFL, in the medium, as expected. (2) As to cytotoxicity, bimodal concentration-dependent cytotoxicity was observed; at 0.1 mg/mL, BEN is not toxic, does not affect in vitro permeability and protects cells from AFB1 toxicity; however, at higher concentrations, it becomes cytotoxic, with IC_50_ values very close to the most effective concentration. (3) When used at 0.1 mg/mL, BEN did not show effects on Caco-2 cells transcriptome. This confirms the interaction between the clay and AFB1 to occur in the medium. (4) By comparing AFB1 and AFB1 + BEN co-exposure data, it is clear that the observed transcriptional changes are due to AFB1 and not to BEN. Furthermore, the most common effect of BEN was to reduce the impact of AFB1 transcriptional effects underneath its toxicity. (5) The most interesting pathways for which BEN showed a protective effect against AFB1 toxic effects are cell integrity, xenobiotic oxidative and conjugative metabolism, afflux- and efflux-transporters, basal metabolism, inflammation and immune response, p53 biological network, apoptosis and carcinogenesis.

Regarding the limits of this study, we did not run RNA-seq investigations in cells exposed to cytotoxic BEN concentrations (above 0.1 mg/mL), alone or in combination with AFB1; this might have revealed possible additive/synergistic effects of the clay. Then, some of our results contradict previously published ones. Possible explanations are the high discrepancies in the range of AFB1 used in many in vitro studies, the differences in the incubation protocols (e.g., pre-treatment) as well as in the clay:AFB1 concentration ratio. It is worth noting that this is one of the few RNA-seq studies involving Caco-2 cells, and the first one investigating the effects against AFB1. Finally, for a deeper understanding of BEN’s positive effects on enterocytes, confirmatory proteomic assays could be envisaged.

## 5. Materials and Methods

### 5.1. Reagents and Chemicals

High glucose Dulbecco’s modified Eagle’s medium (DMEM) with phenol red and L-glutamine (*w*/*o* pyruvate), high glucose DMEM *w*/*o* glutamine and phenol red, fetal bovine serum (FBS), nonessential amino acids (NEAA, 100×) and trypsin 2.5% (10×) were all from Gibco (Life Technologies, Foster City, CA, USA). Penicillin-Streptomycin solution (Penicillin 10,000 IU/mL and Streptomycin 10 mg/mL) was from Biospa (Milan, Italy). Alanine-Glutamine solution (200 mM), EDTA (powder), AFB1 (from *Aspergillus flavus*; CAS Number 1162-65-8), TPA (≥99% purity), NaB (98%), DMSO and Lucifer Yellow CH dilithium salt were purchased from Sigma-Aldrich (St. Louis, MO, USA). Cell proliferation reagent WST-1 was from Roche (Monza, Italy). Qubit™ RNA BR Assay Kit, High Capacity cDNA Reverse Transcription kit and 2X Power SYBR green PCR Master Mix were from Invitrogen (Life Technologies, Foster City, CA, USA) and TapeStation RNA ScreenTape & Reagents from Agilent Technologies (Santa Clara, CA, USA). PET translucent filter inserts 0.4 µM pore size, multi-well plates, and 75 cm^2^ cell culture flasks were purchased from Sarstedt (Verona, Italy).

Bentonite (GLOBALFEED^®^ T1) was kindly provided by Laviosa Chimica Mineraria SpA (Livorno, Italy); the clay was used without thermal or chemical pre-treatment to avoid possible alterations of its physical–chemical characteristics, therefore maintaining the properties described by the producer.

### 5.2. Cell Line

Caco-2 cells (HTB-37™) were purchased from ATCC and were used between passages 24 and 31. Cells were maintained at 37 °C in a humidified atmosphere with 5% CO_2_ in DMEM medium containing phenol red and supplemented with 10% not heat-inactivated FBS, 1% NEAA and 1% Penicillin-Streptomycin solution. Cells were maintained in 75 cm^2^ flasks at a density of 6.4 × 10^5^ cells/flask; the medium was changed every 2 days, and cells were harvested every 7 days using Trypsin-EDTA (0.25–0.2%). Cell number and viability were checked using the trypan blue dye exclusion test. Cell cultures were checked for *Mycoplasma* spp. contamination using the PCR Mycoplasma Test Kit (PromoKine, Heidelberg, Germany).

For all the experiments, cells were grown for 21 days and the medium was changed 3 times a week. Unless otherwise stated, four independent biological replicates (i.e., independent cell culture experiments) were executed in all experiments; in cytotoxicity studies, each concentration was tested in sextuplicate.

All the treatments were performed in a DMEM medium without phenol red and FBS.

### 5.3. 12-O-Tetradecanoylphorbol 13-Acetate and NaB-Mediated Induction of CYP3A4

To increase the expression and activity of CYP3A4, cells were treated with TPA and NaB (100 nM and 4 mM, respectively) for 24 h following the protocol described by Cummins and colleagues [52]. To verify the effect of the pre-treatment, *CYP3A4* mRNA expression was evaluated by quantitative real-time PCR (qPCR), while AFB1 cytotoxicity was assessed by WST-1 Cell Proliferation Reagent.

#### 5.3.1. Quantitative Real-Time PCR

Cells were seeded at a density of 2.1 × 10^5^ cells/well in 6-wells plates; they were grown for 21 days and then treated with NaB and TPA for 24 h. Cells exposed to DMSO 0.25% (vehicle) were used as control.

At the end of the treatment, the monolayer was washed with PBS containing 0.02% EDTA and cells were lysed directly on a plate with 800 µL of RLT buffer (Qiagen, Hilden, Germany) containing 8 μL of β-mercaptoethanol. Samples were vortexed and stored at −80 °C until use.

Total RNA was isolated using the RNeasy mini kit (Qiagen, Hilden, Germany) following the manufacturer’s instructions and quantified by using the NanoDrop 1000 Spectrophotometer (Thermo Fisher Scientific, Waltham, MA, USA).

Complementary DNA (1 μg) was synthesized using the High Capacity cDNA Reverse Transcription kit, following the manufacturer’s instructions. The quantitative real-time PCR amplification was carried out in a final volume of 10 μL, using 2.5 μL of cDNA, the Power SYBR Green PCR Master Mix and the Stratagene MX3000P thermal cycler (Agilent Technologies, Santa Clara, CA, USA) as previously reported [122]. Glyceraldehyde-3-phosphate dehydrogenase (GAPDH) was used as the reference gene, as its expression did not show statistically significant differences between control and treated groups; therefore, the Ct values reported for GAPDH were used for the normalization. Messenger RNA relative quantification (RQ) was performed using the ΔCt method [123]. The list of the target genes and primers [53,124] used for qPCR analyses is reported in Table S7. The experiment was performed in four independent biological replicates.

#### 5.3.2. Cytotoxicity Evaluation

Cells were seeded in 96-well flat-bottom plates at a density of 5 × 10^3^ cells/well and were maintained in culture for 21 days, changing the medium three times a week. After 21 days of cultivation, cells were pre-treated for 24 h with a solution of NaB 4 mM and TPA 100 nM in DMSO 0.25% (IND) or with DMSO 0.25% only (nIND). Afterwards, serial dilutions of AFB1 (0.1, 0.2, 0.5, 5, 15, 30, 60, 90 µM) were prepared in DMEM without FBS (final concentration of DMSO 0.25%). The last AFB1 concentration (90 µM) corresponds to the highest concentration achievable in our conditions without mycotoxin precipitation.

After 48 h of exposure, cell viability was measured using WST-1 Cell Proliferation Reagent following the manufacturer’s instructions with slight modifications. Briefly, at the end of the exposure time, 5 µL (2.5% *v*/*v*) of WST-1 reagent was added to each well. The absorbance was read at 450 nm and 690 nm after 75 min of incubation at 37 °C using the Multiskan™ GO Microplate Spectrophotometer (Thermo Fisher Scientific, Waltham, MA, USA). Results were expressed as the percentage relative to that of cells exposed to the vehicle only with or without induction. The experiment was repeated twice and each concentration was tested in sextuplicate.

### 5.4. Cytotoxicity of BEN and AFB1, Alone or in Combination

The cytotoxicity of BEN was evaluated both in IND and nIND Caco-2 cells. In the first case, a pre-treatment with TPA and NaB was executed (see the detailed protocol in Section 5.3.2). Afterwards, cells were exposed for 48 h to serial dilutions of BEN (0.005, 0.01, 0.03, 0.06, 0.1, 0.2, 0.6 and 1.2 mg/mL) prepared in medium without FBS (0.25% DMSO final concentration). Cells merely exposed to 0.25% DMSO or medium served as controls. Cell viability was measured using WST-1 Cell Proliferation Reagent. Bentonite concentrations were chosen based on the recommended dosage provided by the company and from previous publications [21,40,44,45,125].

Aflatoxin B1 cytotoxicity was estimated in IND cells as described in Section 5.3.2.

In order to determine the BEN concentration that best counteracts the AFB1 effect, cells were treated with a fixed concentration of AFB1 (81 µM) alone or in combination with the different BEN concentrations tested in the cytotoxicity studies. The co-incubation medium was obtained by preparing 10x BEN suspensions and by diluting them 1:10 in 90 µM AFB1 just before the medium was added to the cells. The cytotoxicity was assessed using WST-1 Cell Proliferation Reagent after 48 h of incubation.

### 5.5. Analytical Investigations (LC-MS/MS)

The ability of BEN to bind AFB1 and its metabolites in the medium was assessed by a validated LC-MS/MS method.

AFB1 (81 µM) was incubated alone or in combination with 0.1 mg/mL BEN for 48 h at 37 °C in the dark and in the absence of cells (physical-chemical experiment). Aliquots were collected at the beginning (T0) and the end of the incubation period (T48).

In order to examine the contribution of cellular metabolism and the absorbing power of BEN on AFB1 metabolites, cells cultivated on inserts (biological experiment) were treated following the same experimental conditions. Aliquots were taken at T0 and T48. At T48, the medium was collected from both basolateral and apical compartments. Samples were stored at −80 °C and protected from light until use.

After being thawed at room temperature, samples were vortex-mixed for 30 s and centrifuged at 15,000× *g* for 10 min at 4 °C. Then, 12 µL of the supernatant was diluted into an LC vial with 1.5 mL of a 0.1% formic acid in water:acetonitrile 85:15 (*v*/*v*) solution also containing the internal standard aflatoxin B2 (AFB2); 5 μL were injected in the LC-MS/MS system. Chromatographic separation was achieved with a Waters Acquity UPLC binary pump, equipped with an Acquity BEH C18 (50 × 2.1 mm, 1.7 µm) reversed-phase column, maintained at 40 °C (Waters, Milford, MA, USA). The mobile phase consisted of a variable mixture of 0.1% formic acid in water and acetonitrile under programmed conditions, during a 4 min run at a flow rate of 0.3 mL/min. The LC was coupled to a Waters Xevo TQ-S Micro triple quadrupole mass spectrometer (Waters, Milford, MA, USA), equipped with an electrospray ionization source (ESI) operating in positive mode at a capillary voltage of 3.0 kV. Source and desolvation temperatures were 150 and 600 °C, respectively; desolvation gas flow was 900 L/h and cone gas flow 50 L/h. The two specific transitions that were monitored for each analyte, with the relative cone voltage (CV) and collision energy values (CE) values, were: 313.17 > 241.12 *m*/*z* (CV 80 V; CE 34 eV) and 313.17 > 284.90 *m*/*z* (CV 80 V; CE 20 eV) for AFB1; 329.17 > 273.08 *m*/*z* (CV 70 V; CE 24 eV) and 329.17 > 229.11 *m*/*z* (CV 70 V; CE 38 eV) for AFM1; 297.15 > 141.04 *m*/*z* (CV 78 V; CE 48 eV) and 297.15 > 115.01 *m*/*z* (CV 78 V; CE 50 eV) for AFL; 315.13 > 259.03 *m*/*z* (CV 70 V; CE 26 eV) and 315.13 > 287.06 *m*/*z* (CV 70 V; CE 24 eV) for AFB2. Data acquisition and analysis were performed using MassLynx 4.2 software (Waters, Milford, MA, USA).

### 5.6. Permeability and Trans-Epithelial Electric Resistance Assays

To determine the effect of BEN, alone and in combination with AFB1, on the monolayer integrity, we measured the TEER and the paracellular permeability of LY in cells grown on 12-well inserts [32].

Caco-2 cells were seeded at a density of 6.0 × 10^5^ cells/well and let differentiate for 21 days, changing the medium three times a week. For the maintenance, the medium was removed from the basolateral side first and then from the apical compartment; fresh medium was added following the opposite order.

As to BEN exposure of nIND cells, on the 21st day, the growth medium was substituted with DMEM without phenol red for 24 h; afterward, the medium was removed and cells were treated with 0.01 mg/mL BEN. The clay tested concentration was the one that showed the highest decrease in AFB1 toxicity following the co-incubation studies.

As to co-treatments, on the 21st day, cells were pre-treated as previously described and subsequently incubated with 81 µM AFB1 or 0.1 mg/mL BEN alone or in association. Both IND and nIND cells treated with 0.25% DMSO served as controls.

Before starting the treatment, the monolayer integrity was assessed by measuring the TEER with the EVOM 3 Volthometer (World Precision Instrument, Friedberg, Germany) using the STX-2-PLUS electrode and following the instrument’s instructions. Monolayers with a TEER value > 350 Ω·cm^2^ were considered acceptable [29,126,127]. After 48 h of exposure, monolayers were washed with fresh medium (without FBS) and tested again. The results were expressed as the percentage of the control.

Afterward, paracellular permeability was measured by adding LY at a final concentration of 0.05 mg/mL to the apical compartment and incubating the plate at 37 °C for 90 min [128]. At the end of the incubation period, 150 μL of medium were transferred from the basolateral compartment to a 96-well plate suitable for fluorescence reading. The signal was measured (λex: 428 nm, λem: 540 nm) using VICTOR™X4 Multilabel Plate Reader (Perkin Elmer, Waltham, MA, USA). The experimental design is summarized in Figure S5.

### 5.7. Cells Incubation for Gene Expression Analysis

To evaluate the transcriptional effects of BEN, alone or in combination with AFB1, cells were seeded on 6-well inserts at a density of 2.4 × 10^5^ cells/well [126] and treated as described in Section 5.6. To maintain the same mg/cm^2^ and mg/mL ratios preliminarily used in P96 multi-well plates for cytotoxicity assays, 3.1 or 0.729 mL of medium containing 0.1 mg/mL BEN were used in 6-well and 12-well inserts, respectively.

The trans-epithelial electrical resistance was measured before and after the treatments using the STX-2 electrode. In order to minimize the variability in the monolayer resistance in 6-well inserts, three measurements for each insert were made and then an average value was calculated, as suggested by [32].

Cells were washed with cold PBS, lysed and RNA was extracted following the same protocol described in Section 5.3.1. Total RNA quality was assessed with the TapeStation (Agilent Technologies, Santa Clara, CA, USA). All samples had an RNA Integrity Number (RIN) value > 7.

### 5.8. Whole-Transcriptomic (RNA-seq) Investigations

Gene expression profiles were investigated in all seven experimental conditions. Four independent biological replicates (i.e., independent cell culture experiments) were considered. Thus, a total of 28 tagged RNA-seq libraries were prepared using the Illumina TruSeq Stranded mRNA kit and sequenced on an Illumina NextSeq 500 instrument at the CRIBI NGS Sequencing facility (University of Padua, Padova, Italy) following a 75 bp single-end approach.

#### 5.8.1. Whole Transcriptome Differential Expression Analysis

Raw reads were submitted to preliminary analysis that included counting, quality control (fastQC version 0.11.9; [129]) and trimming (Trimmomatic ver. 0.36; [130]). Reads shorter than 36 bp were excluded, and the ones surviving after trimming were aligned against different ribosomal databases (rfam-5.8s-id98, rfam-5s-id98, silva-arc-16s-id95, silva-arc-23s-id98, silva-bac-16s-id90, silva-bac-23s-id98, silva-euk-18s-id95, silva-euk-28s-id98) using the local sequence alignment tool SortMeRNA (version 4.3.4; [131]) for checking the possible presence of ribosomal RNA (rRNA). Trimmed reads were mapped to the *Homo sapiens* reference genome (GCA_000001405.28, Ensembl release 105) using the software tool STAR (version 2.5.3a; [132]) and following the two-pass mapping mode. The maximum number of mismatches and the maximum number of multiple alignments allowed for a read were set, respectively, to 3 and 10. Gene counts output was used to carry out the differential expression analysis in R studio (R version 4.1.1; [133]) using edgeR package [134]. The transcriptional changes induced by BEN were evaluated, setting the following contrasts: BEN vs. nIND and BEN_IND vs. IND. To investigate the effect of AFB1 and the possible protective effect of the co-treatment with BEN, samples exposed to AFB1 were compared with ones submitted to induction only (AFB1 vs. IND), and cells exposed to co-treatment were compared with ones exposed to AFB1 only (AFB1 + BEN vs. AFB1). The impact of DMSO and TPA + NaB (i.e., the induction) were assessed as well (DMSO vs. nIND; IND vs. nIND).

After filtering out genes expressed at a negligible level or not expressed at all across all libraries (*filterByExpr*), raw counts were normalized by calculating the trimmed mean of M-values (TMM), and data dispersion was estimated (*estimateDisp*). In order to identify DEGs, the quasi-likelihood F-test (*glmQLFTest*) was carried out for all the contrasts highlighted before. A false discovery rate (FDR) ≤ 0.05 and a LFC ≥ 1 were chosen as thresholds of significance. The complete R script used for this analysis is reported in Supplementary File S1.

#### 5.8.2. Functional Enrichment Analysis and Gene Set Enrichment Analysis

To understand which pathways were enriched according to each treatment, DEGs were analyzed with R package ClusterProfiler (version 4.2.1; [135]), specifically using both GO and KEGG over-representation tests (*enrichGO*; *enrichKEGG*).

Finally, the gene set enrichment analysis (GSEA) was performed by means of *ClusterProfiler* package. A list of genes produced by the *glmQLFTest* function was used to create the input file for this computational analysis, pre-ranking all genes according to their *p*-value using “1-pvalue” and “−(1-pvalue)” to include the direction of their expression in the analysis (up- or down-regulation, respectively).

### 5.9. Statistical Analyses

Sigmoidal dose–response curves and the histograms reported in the paper were obtained using GraphPad Prism software (version 5, San Diego, CA, USA). As to dose–response curves, a non-linear regression (log(inhibitor) vs. normalized response, variable slope) was built. The half-maximal inhibitory concentration and the R^2^ were provided by the software.

Quantitative real-time PCR results and data of permeability and TEER were analyzed with Mann–Whitney U-test when two groups were considered, while when more groups were tested, we used one-way ANOVA, setting the level of significance to *p* < 0.05.

## Data Availability

Raw Illumina sequencing data have been deposited in GenBank (SRA) under the BioProject accession PRJNA843983.

## Supplementary Materials

The following are available online at https://www.mdpi.com/article/10.3390/toxins14070435/s1, Figure S1: Effects of the *CYP3A4* induction protocol on differentiated Caco-2 cells; Figure S2: Dose–response curves of BEN and AFB1 in differentiated Caco-2 cells after 48 h of incubation; Figure S3: Effect of 0.1 mg/mL BEN on TEER and LY dye paracellular permeability in nIND Caco-2 cells; Figure S4: Effect of 0.1 mg/mL BEN, AFB1, and the combination BEN + AFB1 on LY dye uptake in *CYP3A4*-induced Caco-2 cells (IND); Figure S5: Scheme of the experimental design for TEER evaluation; Table S1: BEN adsorbing capacity toward aflatoxin AFB1, AFM1 and AFL, and its effects on AFB1, AFM1 and AFL transport rate; Table S2: Sequencing and mapping results; Table S3: Differential expression analysis; Table S4: GO over-representation analysis; Table S5: GSEA analysis; Table S6: KEGG over-representation analysis; Table S7: Target genes and primers used for the quantitative real-time PCR analysis; File S1: R code used for Differential Expression Analysis, Functional Analysis and graphs drawing.
