# Supplementary material for: Does Bentonite Cause Cytotoxic and Whole-Transcriptomic Adverse Effects in Enterocytes When Used to Reduce Aflatoxin B1 Exposure?
Source: Toxins (Basel). 2022 Jun 26;14(7):435. doi: 10.3390/toxins14070435 (PMC9322703; doi:10.3390/toxins14070435)
Supplement: Supplementary file 1 [file toxins-14-00435-s001.zip › Mucignat & Bassan - SplOnline File S1 Rscript.pdf]

FileS1. R code used for Differential Expression Analysis, Functional Analysis and graphs drawing

→*Differential Expression Analysis (EdgeR)*

Import dataset, read it and define groups

```
>library (edgeR)
>input <-read.delim ("rawreads.txt" , header = TRUE, sep = "",
stringsAsFactors = FALSE, row.names = "GENEID")
>targets<- read.delim ("targets.txt")
>group <-factor (paste (targets$CONDITION))
```

Create a list-based data object called DGEList and filter raw data

```
>dge <- DGEList (counts= input, group = group)
>keep<-filterByExpr(dge, group=group)
>filter<- dge[keep, ,keep.lib.sizes=FALSE]
```

Normalization of the libraries sizes

```
>norm.dge <-calcNormFactors (filter)
```

Cpm and lcpm and plot of the library sizes post-filtering and normalization

```
>cpm_norm <- cpm(norm.dge)
>logcpm_norm <- cpm(norm.dge, log=TRUE)
```

Set study design

```
>design <- model.matrix(~ 0 + group, data=norm.dge$samples)
>colnames (design) <- levels(norm.dge$samples$group)
```

Set contrasts

```
>mycontrasts <- makeContrasts (IND=IND-DMSO, BEN_IND=BEN_IND-IND,
AFLA=AFLA-IND, BEN_AFLA=AFLA_BEN-AFLA, levels= design)
```

Estimate dispersion and relative plot

```
>disp <- estimateDisp(norm.dge, design, robust=TRUE)
>plotBCV (disp, main ="Dispersion")
```

MDS Plot

```
>colors <- c ("red", "violetred4","orange","green","blue")
>shape <- c(18,8,15,17,16)
>plotMDS(norm.dge, col= colors[group],pch= shape[group], xlim=c(-3,+3),
ylim=c(-2, +2))
>legend("topleft",legend = levels(group),  col= colors, pch= shape)
```

→*Quasi-likelihood F-tests*

1. IND vs DMSO

```
>fit <- glmQLFit(disp, design, robust=TRUE)
>QLFtest <- glmQLFTest(fit_qlf,contrast = mycontrasts[,"IND"])
>topTags(QLFtest, n = Inf, sort.by = "p.value",adjust.method = "fdr")
>is.de<- decideTestsDGE(QLFtest)
>summary (is.de)
```

2. BEN IND vs IND

```
>fit <- glmQLFit(disp, design, robust=TRUE)
>QLFtest <- glmQLFTest(fit,contrast = mycontrasts[,"BEN_IND"])
>topTags(QLFtest, n = Inf, sort.by = "p.value",adjust.method = "fdr")
>is.de<- decideTestsDGE(QLFtest)
>summary (is.de)
```

3. AFB1 vs IND

```
>fit <- glmQLFit(disp, design, robust=TRUE)
>QLFtest <- glmQLFTest(fit,contrast = mycontrasts[,"AFB1"])
>topTags(QLFtest, n = Inf, sort.by = "p.value",adjust.method = "fdr")
>is.de<- decideTestsDGE(QLFtest)
>summary (is.de)
```

4. AFB1+BEN vs AFB1

```
>fit <- glmQLFit(disp, design, robust=TRUE)
>QLFtest <- glmQLFTest(fit,contrast = mycontrasts[,"AFB1+BEN"])
>topTags(QLFtest, n = Inf, sort.by = "p.value",adjust.method = "fdr")
>is.de<- decideTestsDGE(QLFtest)
>summary (is.de)
```

The same parameters were used also for the analysis of the following contrasts: BEN vs nIND, DMSO vs nIND. For these last two contrasts it wasn’t conducted a functional analysis because there weren’t DEGs.

→*Functional analysis (clusterProfiler)*

```
>library (clusterProfiler)
>library (enrichplot)
>library (org.Hs.eg.db)
>library (pathview)
>library (DOSE)
>library (ggplot2)
>library (viridis)
```

A. GO over-representation test

1.Data input (DEGs and LogFC)

```
>x <-read.csv2 ("input.GO.csv",header =FALSE)
>geneList <- x[,2]
>names(geneList)<-as.character (x[,1])
```

```
>geneList <-sort (geneList, decreasing=TRUE)
>DEGS <-names (geneList)
>back<-topTags(QLFtest, n=Inf)$table
>backlist <-row.names (back)
```

2.GO over-representation test

```
>enrich <-enrichGO (gene=DEGS, OrgDb= org.Hs.eg.db, keyType= "ENSEMBL",
ont="BP",minGSSize=10, maxGSSize=500, pvalueCutoff= 0.05, qvalueCutoff=
0.2, pAdjustMethod= "fdr", universe= backlist, readable= TRUE,
pool=FALSE)
>simple <- simplify(enrich, cutoff=0.7, by ="p.adjust", select_fun=min)
```

B. KEGG over-representation test

1. Data input

```
>x <-read.csv2 ("input.KEGG.csv”,header =FALSE)
>geneList <- x[,2]
>names(geneList)<-as.character (x[,1])
>geneList <-sort (geneList,decreasing=TRUE)
>DEGS <-names (geneList)
>back<-topTags(QLFtest, n=Inf)$table
>backlist <-row.names (back)
```

2. Convert ENSEMBL gene IDs to ENTREZ IDs

```
>DEGS.entrez<- bitr(DEGS, fromType = "ENSEMBL", toType = c("ENTREZID"),
OrgDb = "org.Hs.eg.db")
>backlist.entrez<- bitr(backlist, fromType = "ENSEMBL", toType =
c("ENTREZID"),OrgDb = "org.Hs.eg.db")
>DEGS.kegg<- DEGS.entrez$ENTREZID
>backlist.kegg<-backlist.entrez$ENTREZID
```

3. KEGG over-representation test and relative plots

```
>enrichKEGG<-enrichKEGG(DEGS.kegg,organism = "hsa", keyType =
"kegg",pvalueCutoff = 0.05, pAdjustMethod = "BH", universe=backlist.kegg,
minGSSize = 10, maxGSSize = 500, qvalueCutoff = 0.2, use_internal_data =
FALSE)
```

C. GO GSEA

1. Input data

```
>inputGSEA<- read.csv2("inputGSEA.csv", header=FALSE)
>geneList_GSEA <- as.numeric (inputGSEA [,2])
>names(geneList_GSEA) <- as.character(inputGSEA [,1])
>geneList_GSEA <- sort(geneList_GSEA, decreasing = TRUE)
```

2. GSEA

```
>gsea <- gseGO(geneList=geneList_GSEA,
                      ont ="BP",
```

```
                         keyType = "ENTREZID",
                         minGSSize = 10,
                         maxGSSize = 500,
                         pvalueCutoff = 0.05,
                         verbose = TRUE,
                         eps = 0,
                         OrgDb = org.Hs.eg.db,
                         pAdjustMethod = "BH",
                         by="fgsea")
```

```
>simple.gsea<- simplify(gsea, cutoff=0.7, by ="p.adjust", select_fun=min)
>dotplot(simple.gsea, showCategory=20, split=".sign", label_format= 30,
orderBy="x") + facet_grid(.~.sign)
```

With the same parameters the analysis was conducted on all the contrasts changing the input data; for GO enrichment analysis of AFB1 vs IND and AFB1+BEN vs AFB1 the input was divided between up-regulated and down-regulated genes.

Graphs

Barplot KEGG enrichment analysis AFB1 vs IND

```
>barplot(enrichKEGG, showCategory=15)+ ggtitle ("Barplot for KEGG
enrichment analysis")
```

Barplot GO enrichment analysis of up-regulated genes of AFB1 vs IND

```
>barplot (simple, showCategory=20) + ggtitle ("Barplot for GO enrichment
analysis")
```

Dotplot GO GSEA AFB1+BEN vs AFB1

```
>dotplot (simple.gsea, showCategory=10, split=".sign", label_format= 40,
orderBy="x") + facet_grid(.~.sign)+ggtitle("Dotplot for GO pathway GSEA")
```

Barplot GO enrichment analysis of down-regulated DEGs of AFB1+BEN vs AFB1

```
>barplot (simple, showCategory=20)+ggtitle("Barplot for GO enrichment
analysis")
```

D. Functional analysis of the 2018 DEGs in common between AFB1+BEN vs AFB1 and AFB1 vs IND

GO over-representation test

```
>geneList <-read.csv2 ("inputGO.match.csv",header =FALSE)
>DEGS <-geneList[,1]
>back<-topTags(qlfBENafla, n=Inf)$table
>backlist <-row.names (back)


>enrich.match <-enrichGO (gene=DEGS, OrgDb= org.Hs.eg.db, keyType=
"ENSEMBL", ont="BP",minGSSize=10, maxGSSize=500, pvalueCutoff= 0.05,
qvalueCutoff= 0.2, pAdjustMethod= "fdr", universe= backlist, readable=
TRUE, pool=FALSE)
>simple.match <-simplify(enrich.match, cutoff=0.7, by ="p.adjust",
select_fun=min)
```

Graph

```
>barplot (simple.match, showCategory=20)+ggtitle("Barplot for GO
enrichment analysis")
```

→Heatmap

```
>x <- read.delim("input_logCPMheatmapDEGS.txt",row.names="Gene",
stringsAsFactors=FALSE, header = TRUE)
>library("pheatmap")
>library("RColorBrewer")
>data <- x
>basedir <- getwd()
>maxclust <- 4
>col.pal <- brewer.pal(11,"RdYlGn")
>drows1 <- "euclidean"
>dcols1 <- "euclidean"
>filename <- "my.logCPMpheatmap.tiff"
>outfile <- paste(basedir, filename, sep="/")
>hm.parameters <- list(data,
                       color = col.pal,
                       cellwidth = 15, cellheight = 12, scale = "none",
                       treeheight_row = 200,
                       kmeans_k = NA,
                       show_rownames = T, show_colnames = T,
                       main = "DEGs of interest",
                       clustering_method = "average",
                       cluster_rows = FALSE, cluster_cols = TRUE,
                       clustering_distance_rows = drows1,
                       clustering_distance_cols = dcols1,
                       width=12, heigh=13)
>do.call("pheatmap", c(hm.parameters, filename=outfile))
```
