# Supplementary material for: Does Bentonite Cause Cytotoxic and Whole-Transcriptomic Adverse Effects in Enterocytes When Used to Reduce Aflatoxin B1 Exposure?
Source: Toxins (Basel). 2022 Jun 26;14(7):435. doi: 10.3390/toxins14070435 (PMC9322703; doi:10.3390/toxins14070435)
Supplement: Supplementary file 1 [file toxins-14-00435-s001.zip › Mucignat & Bassan - SplOnline.pdf]

toxins

Supplementary materials

Does Bentonite Cause Cytotoxic and Whole-Transcriptomic Adverse Effects in Enterocytes When Used to Reduce Aflatoxin B1 Exposure?

Greta Mucignat [1,§], Irene Bassan [1,§], Mery Giantin [1], Marianna Pauletto [1], Anisa Bardhi [2], Silvia Iori [1], Rosa M. Lopparelli [1], Andrea Barbarossa [2], Anna Zaghini [2], Enrico Novelli [1] and Mauro Dacasto [1,*]

[1] Department of Comparative Biomedicine and Food Science, University of Padua, viale dell'Università 16, I-35020 Agripolis Legnaro (Padua), Italy; greta.mucignat@unipd.it (G.M.); irene.bassan@unipd.it (I.B.); mery.giantin@unipd.it (M.G.); marianna.pauletto@unipd.it (M.P.); silvia.iori@phd.unipd.it (S.I.); rosa.lopparelli@unipd.it (R.M.L.); enrico.novelli@unipd.it (E.N.)

[2] Department of Veterinary Medical Sciences, *Alma Mater Studiorum* University of Bologna, via Tolara di Sopra 50, I-40064 Ozzano dell'Emilia (Bologna), Italy; anisa.bardhi@unibo.it (A.B.); andrea.barbarossa@unibo.it (A.B.); anna.zaghini@unibo.it (A.Z.)

* Correspondence: mauro.dacasto@unipd.it (M.D.); Tel.: +39-049-827-2935

§ These authors contributed equally to this work

FIGURES

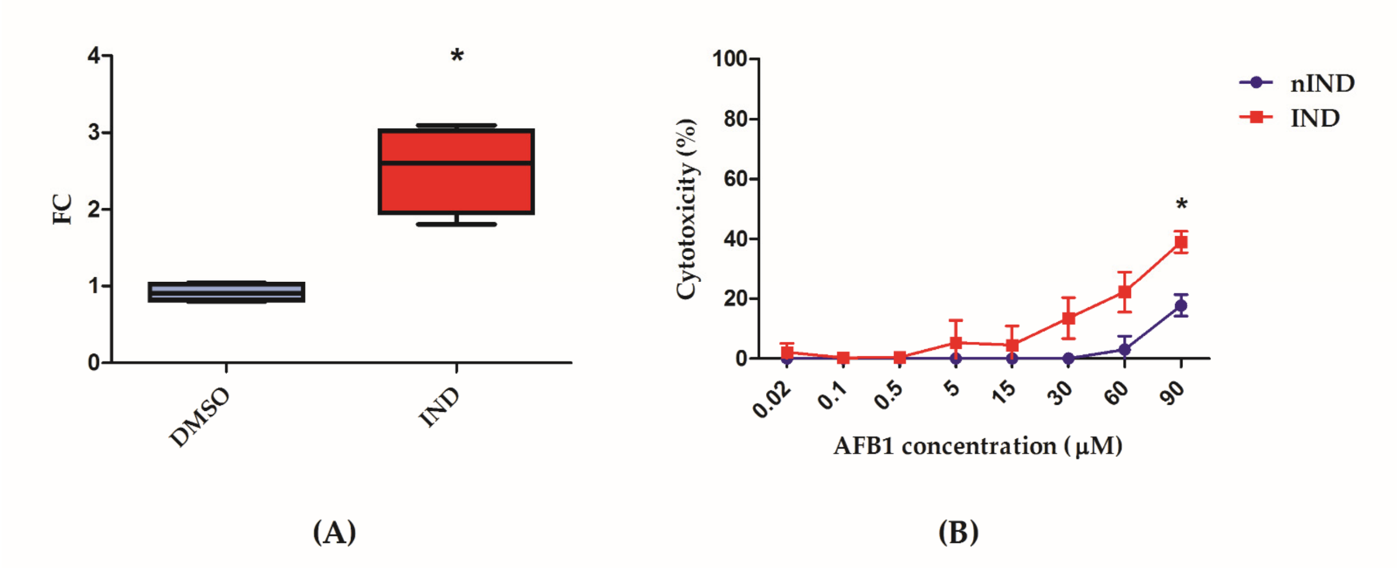

Figure S1. Effects of the cytochrome P450 3A4 (*CYP3A4*) induction protocol (see section 5.3. of Materials and Methods), on differentiated Caco-2 cells. **(A)** Basal CYP3A4 mRNA levels (quantitative real-time PCR) in dimethylsufoxide (DMSO) or induced (IND) Caco-2 cells. **(B)** Aflatoxin B1 (AFB1) cytotoxicity (WST-1) in non-induced (nIND, blue) and IND (red) Caco-2 cells. Cells were exposed to increasing concentrations of AFB1 for 48 h. Data are expressed as mean Fold Change (FC) ± mean standard error (SEM) of two independent cell culture experiments, each one run in sextuplicate. *: $P < 0.05$ (Mann-Whitney U-test).

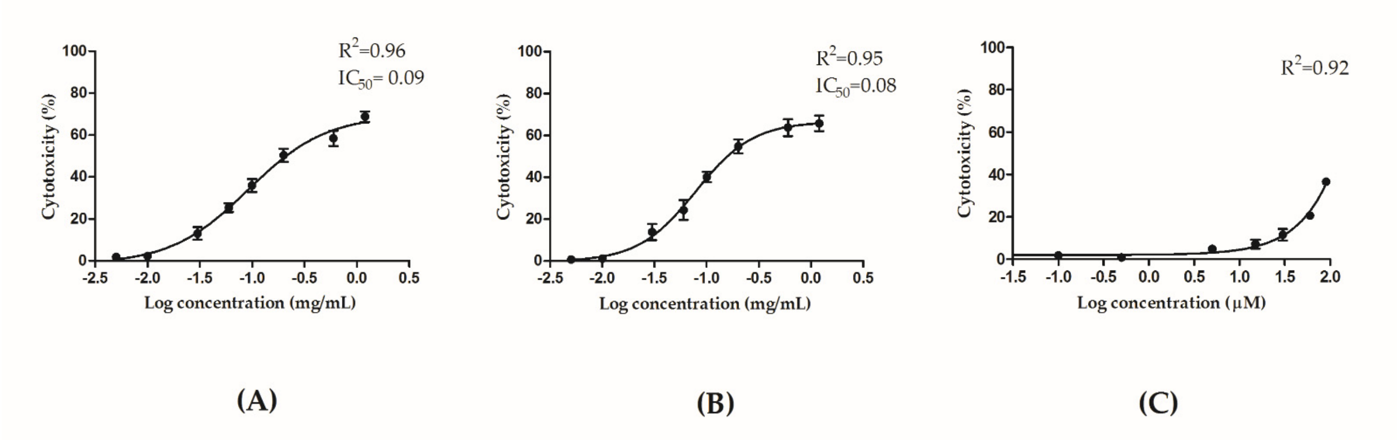

Figure S2. Dose-response curves of bentonite (BEN; **A**, **B**) and aflatoxin B1 (AFB1; **C**) in differentiated Caco-2 cells after 48 h of incubation. As to BEN, cells were previously induced (IND, **A**) or non-induced (nIND, **B**) by using the protocol mentioned in section 5.3. of Materials and Methods. However, only IND cells were used to assess the AFB1 cytotoxicity (**C**). The corresponding half-maximal inhibitory concentration (IC_{50}) and R^2 are reported, too. Data are expressed as the mean cytotoxicity rate ± mean standard error (SEM) of four independent cell culture experiments, each one run in sextuplicate.

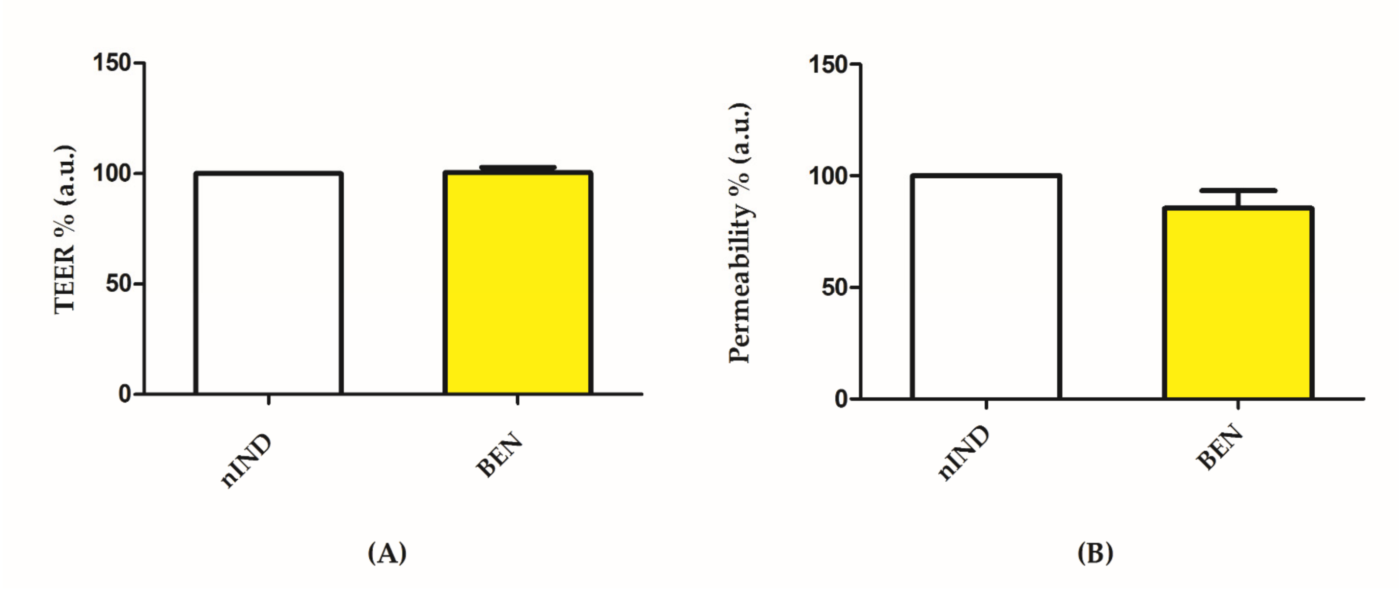

Figure S3. Effect of 0.1 mg/mL bentonite (BEN) on trans-epithelial electrical resistance (TEER, **A**) and Lucifer Yellow (LY) dye paracellular permeability **(B)** in not induced (nIND) Caco-2 cells. Data (five independent cell culture experiments, each one run in duplicate) are reported as mean percentage ± mean standard error (SEM) of control cells (nIND), whose value was set at 100%.

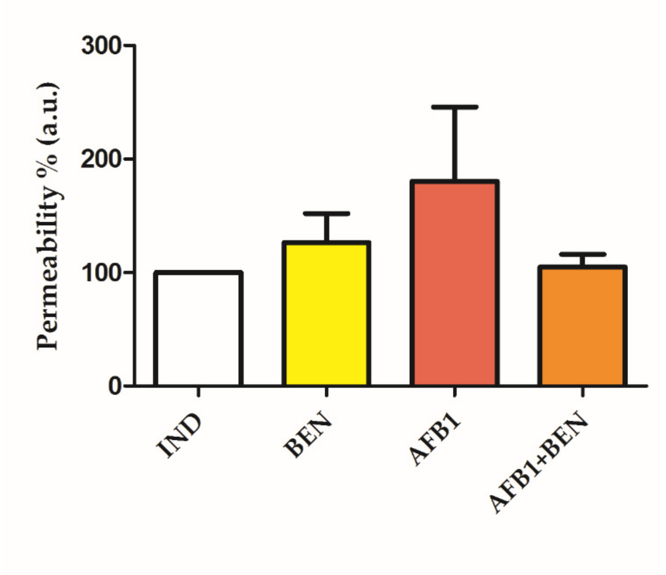

Figure S4. Effect of 0.1 mg/mL bentonite (BEN), aflatoxin B1 (AFB1, and the combination AFB1+BEN on the Lucifer Yellow (LY) dye uptake in cytochrome P450 3A4 (*CYP3A4*)-induced Caco-2 cells (IND). Data (five independent cell culture experiments, each one run in duplicate) are reported as mean percentage ± mean standard error (SEM) of control cells (IND), whose value was set at 100%.

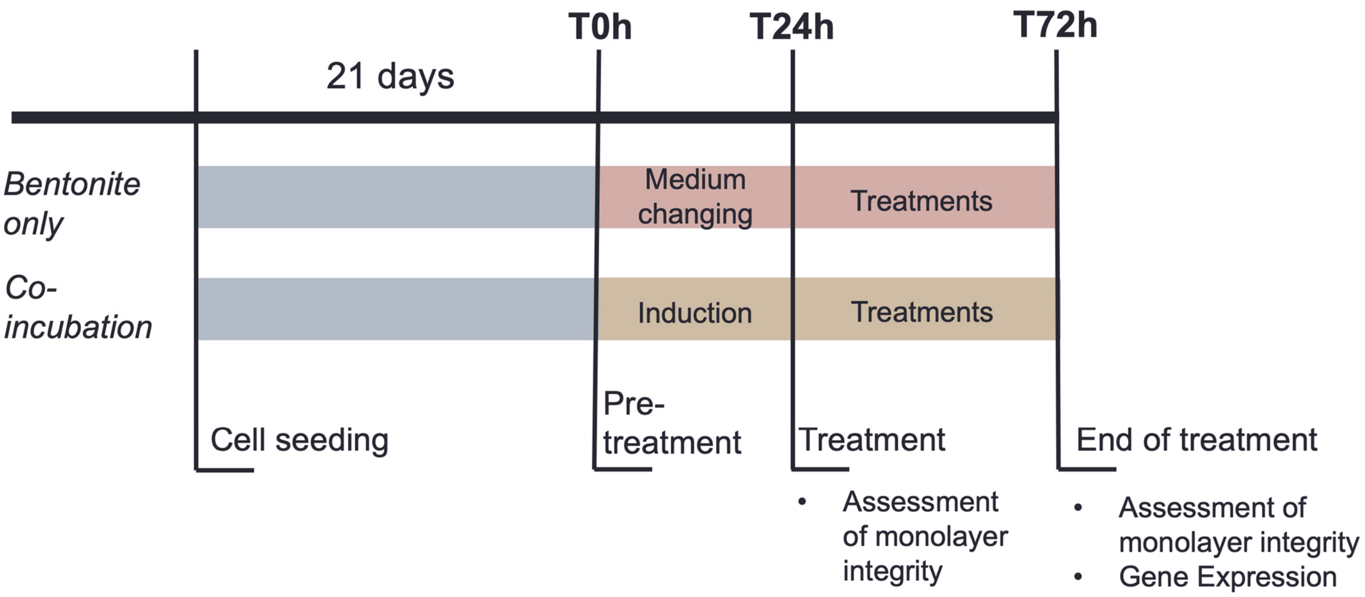

Figure S5. Scheme of the experimental design for TEER evaluation.

TABLES

Table S1. Bentonite (BEN) adsorbing capacity toward aflatoxin B1 (AFB1), AFM1 and aflatoxicol (AFL) (**A**), and its effects on AFB1, AFM1 and AFL transport rate (**B**). Induced (IND) Caco-2 cells, cultivated in inserts, were exposed either to 0.1 mg/mL BEN with 81 μM AFB1 (AFB1+BEN) or to 81 μM AFB1 (AFB1). After 48 h of incubation at 37°C, AFB1, AFM1 and AFL amounts were measured using set uup and validated LC-MS/MS protocols. In (A), the percentages of total free analytes present in medium containing AFB1+BEN (AFB1+BEN) are compared to those detected in the medium containing only AFB1 (AFB1). In (**B**), the percentages of total free analytes that passed into the basolateral compartment (considering negligible the amount of analytes accumulating in the cell) are reported. IND cells were exposed to the combination AFB1+BEN, and the total free analyte (AFB1 amount not adsorbed by BEN) was quantified. Data are reported as mean percentage ± mean standard error (SEM) of (five independent cell culture experiments, each one run in duplicate).

**A**	**Adsorbance** (AFB1+BEN) / AFB1					
	**AFB1**		**AFM1**		**AFL**	
	57.6% ± 2.7%		65.2% ± 2.5%		50.3% ± 2.4%	
**B**	**Transport rate**					
	**AFB1**		**AFM1**		**AFL**	
	AFB1	AFB1+BEN	AFB1	AFB1+BEN	AFB1	AFB1+BEN
	44.0% ± 0.7%	44.3% ± 0.5%	28.7% ± 2.3%	26.3% ± 1.7%	45.0% ± 1.6%	45.5% ± 1.0%

Table S2. Sequencing and mapping results. The table reports the sequenced RNA-seq libraries, including for each of them: i) the number of raw reads obtained; ii) the number of reads after trimming; iii) the number of mapped reads and the corresponding percentage.

Sample	Nr. raw reads	Nr. reads after trimming	Nr. reads mapping	% of reads mapping
nIND_1	38,753,412	38,747,397	34,202,477	88.27%
nIND_2	34,556,667	34,551,831	30,595,113	88.55%
nIND_3	34,880,649	34,876,370	30,875,497	88.53%
nIND_4	30,730,396	30,725,991	27,165,512	88.41%
BEN_1	34,875,341	34,869,049	30,801,753	88.34%
BEN_2	34,064,315	34,059,671	29,943,504	87.91%
BEN_3	32,400,296	32,394,135	28,498,424	87.97%
BEN_4	33,908,517	33,903,416	30,114,514	88.82%
DMSO_1	76,293,408	76,278,463	66,969,400	87.80%
DMSO_2	30,358,539	30,354,031	26,877,467	88.55%
DMSO_4	32,428,381	32,421,823	28,622,990	88.28%
IND_1	36,152,588	36,142,004	31,934,024	88.36%
IND_2	33,948,084	33,937,041	30,243,757	89.12%
IND_3	33,591,422	33,575,594	29,847,251	88.90%
IND_4	31,528,849	31,517,526	28,170,407	89.38%
AFB1_1	30,707,453	30,701,594	27,088,357	88.23%
AFB1_2	32,188,544	32,182,449	28,594,762	88.85%
AFB1_3	30,036,340	30,031,458	26,945,481	89.72%
AFB1_4	27,952,248	27,946,192	25,204,321	90.19%
BEN_IND_1	30,236,385	30,230,200	26,888,452	88.95%
BEN_IND_2	31,085,810	31,080,715	27,647,275	88.95%
BEN_IND_3	31,595,350	31,590,298	28,267,300	89.48%
BEN_IND_4	32,653,312	32,647,356	28,982,635	88.77%
AFB1+BEN_2	32,334,463	32,327,980	28,924,394	89.47%
AFB1+BEN_3	38,562,827	38,552,898	34,663,550	89.91%
AFB1+BEN_4	32,091,773	32,084,972	28,901,722	90.08%
**Mean**	34,535,207	34,528,094	30,652,705	88.84%

Table S7. Target genes and primers used for the quantitative real-time quantitative PCR analysis.

Target gene	Forward primer	Reverse primer	Reference
CYP3A4	5′-CATTCCTCATCCCAATTCTTGAAGT-3′	5′-CCACTCGGTGCTTTTGTGTATCT-3′	[53]
GAPDH	5′-CTCTGCTCCTCCTGTTCGAC-3′	5′-ACGACCAAATCCGTTGACTC-3′	[125]
